# Halvade somatic: Somatic variant calling with Apache Spark

**DOI:** 10.1093/gigascience/giab094

**Published:** 2022-01-12

**Authors:** Dries Decap, Louise de Schaetzen van Brienen, Maarten Larmuseau, Pascal Costanza, Charlotte Herzeel, Roel Wuyts, Kathleen Marchal, Jan Fostier

**Affiliations:** IDLab, Ghent University - imec, Technologiepark 126, B-9052 Ghent, Belgium; IDLab, Ghent University - imec, Technologiepark 126, B-9052 Ghent, Belgium; IDLab, Ghent University - imec, Technologiepark 126, B-9052 Ghent, Belgium; Intel, Veldkant 31, B-2550 Kontich, Belgium; imec, Kapeldreef 75, B-3001 Leuven, Belgium; imec, Kapeldreef 75, B-3001 Leuven, Belgium; IDLab, Ghent University - imec, Technologiepark 126, B-9052 Ghent, Belgium; IDLab, Ghent University - imec, Technologiepark 126, B-9052 Ghent, Belgium

**Keywords:** Apache Spark, somatic variant calling, GATK/Mutect2, Strelka2

## Abstract

**Background:**

The accurate detection of somatic variants from sequencing data is of key importance for cancer treatment and research. Somatic variant calling requires a high sequencing depth of the tumor sample, especially when the detection of low-frequency variants is also desired. In turn, this leads to large volumes of raw sequencing data to process and hence, large computational requirements. For example, calling the somatic variants according to the GATK best practices guidelines requires days of computing time for a typical whole-genome sequencing sample.

**Findings:**

We introduce Halvade Somatic, a framework for somatic variant calling from DNA sequencing data that takes advantage of multi-node and/or multi-core compute platforms to reduce runtime. It relies on Apache Spark to provide scalable I/O and to create and manage data streams that are processed on different CPU cores in parallel. Halvade Somatic contains all required steps to process the tumor and matched normal sample according to the GATK best practices recommendations: read alignment (BWA), sorting of reads, preprocessing steps such as marking duplicate reads and base quality score recalibration (GATK), and, finally, calling the somatic variants (Mutect2). Our approach reduces the runtime on a single 36-core node to 19.5 h compared to a runtime of 84.5 h for the original pipeline, a speedup of 4.3 times. Runtime can be further decreased by scaling to multiple nodes, e.g., we observe a runtime of 1.36 h using 16 nodes, an additional speedup of 14.4 times. Halvade Somatic supports variant calling from both whole-genome sequencing and whole-exome sequencing data and also supports Strelka2 as an alternative or complementary variant calling tool. We provide a Docker image to facilitate single-node deployment. Halvade Somatic can be executed on a variety of compute platforms, including Amazon EC2 and Google Cloud.

**Conclusions:**

To our knowledge, Halvade Somatic is the first somatic variant calling pipeline that leverages Big Data processing platforms and provides reliable, scalable performance. Source code is freely available.

## Introduction

Somatic mutations are changes in the DNA of a cell that are introduced during the lifetime of a living organism. Owing to their role in the development of cancer, the accurate detection of somatic variants is of key importance. The broad landscape of somatic variants has been characterized by large-scale research projects such as The Cancer Genome Atlas Program (TCGA) [1], The Cancer Cell Line Encyclopedia [2], and the International Cancer Genome Consortium [3]. In clinical practice, the profiling of genomic variants and signatures in tumors is increasingly adopted to provide patient-tailored therapies.

Cancer mutations are often characterized using next-generation sequencing (NGS) technology. In a typical setting, a tumor sample is accompanied by a matched normal sample from which germline variants are determined. Cancer-specific mutations are those that are present in the tumor sample but absent from the normal sample. The tumor sample is often heterogeneous: it may contain different subpopulations of cancer cells with distinct molecular signatures [4]. As such, mutations can appear in a bulk tumor sample with varying frequency. To also capture low-frequency variants, a high sequencing depth of the tumor sample is warranted (typically ≥50×) [5]. Together with the sequencing of the matched normal sample, this gives rise to large volumes of raw sequencing data, especially for whole-genome sequencing (WGS). In turn, this leads to high processing times. To illustrate this, we consider the variant calling pipeline according to the GATK best practices recommendations [6] that uses BWA [7] for read mapping, Picard [8] and GATK [9] for data preprocessing, and Mutect2 [10] for somatic variant calling. To process an Illumina HiSeq 2000 WGS dataset of the HCC1395 sample (breast cancer cell line) with a sequencing depth of 62× (tumor) and 34× (normal) and using a 36-core machine (dual 2.30 GHz Intel Xeon Gold 6140 CPU with 196 GB of RAM), we measured a runtime of ∼3.5 days (∼84.5 h): ∼13 h for read mapping, ∼41 h for data preprocessing, and ∼30.5 h for variant calling. This very high runtime is caused not only by the large volume of input sequencing data to process (∼693 GB uncompressed) but also due to the fact that Picard, GATK, and Mutect2 do not efficiently make use of modern, multi-core architectures because most of their codebase is single-threaded. As such, the computational resources provided by modern compute systems are underutilized.

**Figure 1:**
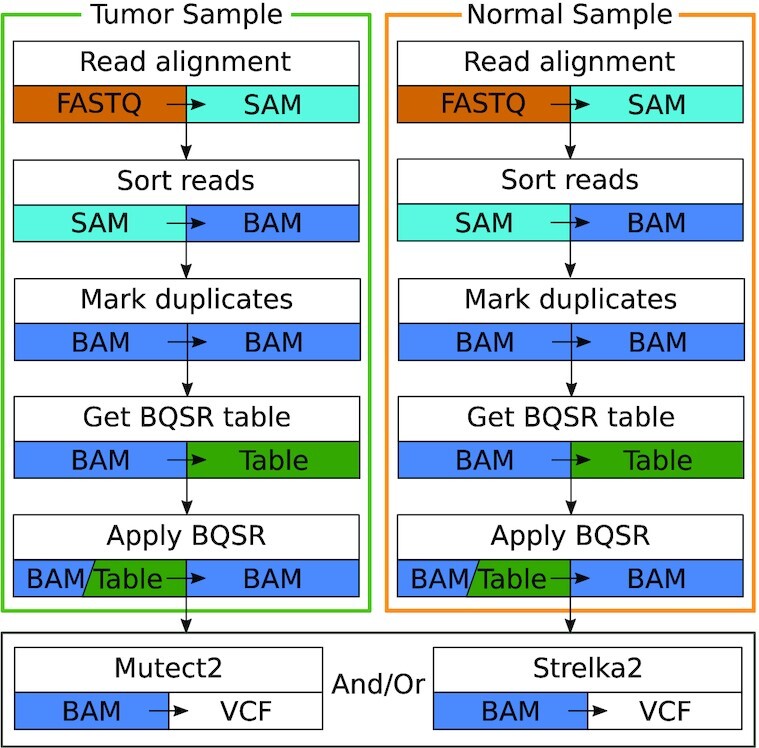
: Somatic variant calling pipeline implemented in Halvade Somatic. Strelka2 can be run as an alternative or complementary tool to Mutect2.

We present Halvade Somatic, a scalable software framework that leverages Apache Spark [11] to efficiently perform somatic variant calling using multi-node and/or multi-core compute platforms. Halvade Somatic creates and manages parallel data streams that are processed by multiple instances of existing tools on different CPU cores. It implements the somatic variant calling pipeline according to the GATK best practices recommendations (see Fig. 1 for an overview). Next to Mutect2 [10], Strelka2 [12] is supported as an alternative or complementary variant calling tool. Both Mutect2 and Strelka2 use an algorithm that models joint allele frequencies to call somatic variants [13], and both tools have been widely adopted by the scientific community. The support for both Mutect2 and Strelka2 allows for consensus variant calling by combining the results of both tools, a commonly used practice that yields more robust results. To distribute the workload in smaller subtasks, Halvade Somatic uses the same general principles as its predecessors that were designed for germline variant calling from DNA and RNA sequencing data [14,15]: (i) the read alignment step can be parallelized by read; i.e., the process of aligning a particular read is independent of the alignment of another read; and (ii) preprocessing and variant calling steps are parallelized by genomic region; e.g., calling somatic variants in a particular genomic region is independent of variant calling in other regions. Compared with its counterparts for germline variant calling, Halvade Somatic is significantly more complex. First, the volume of data to process is larger owing to the presence of 2 samples (tumor + normal) instead of only a single sample in the case of germline variant calling. The data of both samples must be partitioned in a consistent manner across the parallel compute tasks while maintaining good load balance. Second, to have a good concordance between somatic variants called by the original (sequential) pipeline and the variants called by Halvade Somatic, we found that a careful design of the parallel base quality score recalibration (BQSR) step was essential: whereas BQSR for the germline variant calling pipeline could simply be applied to different genomic regions independently, the construction of *genome-wide* recalibration tables appears essential for somatic variant calling. Because of this, additional communication steps are required to aggregate locally computed, partial BQSR statistics into global statistics. Finally, whereas Halvade for germline variant calling was based on the MapReduce framework [16], Halvade Somatic is a re-implementation from scratch that leverages the Spark framework. Compared with MapReduce, Spark offers a richer framework with support for more complex communication and synchronization primitives, as well as the ability to keep data in memory. As such, Spark is much better suited to deal with the different communication steps that arise from the parallelization of somatic variant calling pipelines.

Halvade Somatic is highly efficient: using a single 36-core compute node, runtime for the GATK/Mutect2 pipeline is reduced from ∼84.5 to ∼19.5 h. This speedup of 4.3  times originates from a better utilization of the same hardware resources. Scaling to 16 nodes further reduces runtime to ∼1 h 21 min, an additional speedup of ∼14.4 times, i.e., a total speedup of ∼62.4 times over the original pipeline. Users can select between the Mutect2 or Strelka2 variant callers or can choose to execute both tools, thus generating 2 separate variant callsets that can be combined and filtered to obtain high-confidence consensus variants [17]. Variant calling from both WGS as well as whole-exome sequencing (WES) data is supported. To facilitate the execution of Halvade Somatic on a workstation without Spark installation, we provide a Docker image. Halvade Somatic can be executed on a wide variety of compute platforms, including the Amazon EC2 and Google Cloud.

## Positioning with respect to state of the art

As reviewed in [18], many bioinformatics workflows have been accelerated using Hadoop MapReduce or Spark. Tools such as BigBWA [19], SEAL [20], and Halvade [14] rely on the MapReduce programming model to accelerate sequence analysis pipelines. Whereas BigBWA and SEAL focus primarily on the read mapping phase, Halvade leverages MapReduce to accelerate an end-to-end germline GATK-based variant calling pipeline. The combination of parallel processing, the distributed-memory sorting functionality of Hadoop MapReduce, and a scalable storage solution such as the Hadoop Distributed File System (HDFS) [21] yield efficient workflows that strongly reduce runtime. A more complex pipeline for germline variant calling from RNA-sequencing data was implemented in Halvade-RNA [15]. Halvade-RNA requires 2 successive MapReduce jobs to express the workflow. In between both MapReduce jobs, large volumes of intermediate data are stored on disk and loaded again when the second job commences.

The stringent map-sort-reduce paradigm as well as its disk-oriented processing are the main drawbacks of Hadoop MapReduce. The introduction of Spark solved these shortcomings and led to the introduction of a new generation of sequence analysis pipelines. SparkBWA [22] and StreamBWA [23] leverage Spark for the task of read mapping, whereas SparkGA [24,25] implements a more comprehensive pipeline for germline variant calling according to the GATK best practices recommendations. A Spark-based adaption of an RNA-seq variant calling pipeline was provided by SparkRA [26].

These MapReduce and Spark-based workflows have in common that they execute, in parallel, multiple instances of *existing* tools (e.g., BWA [7] or GATK [9]) on subsets of the data. In other words, MapReduce and Spark are used (i) to provide scalable I/O; (ii) to manage parallel data streams; and (iii) for task scheduling, synchronization, and communication purposes. Most of the actual processing of sequencing data is done by existing tools. This modular approach makes it easy to integrate newer versions of these tools or to switch between alternative tools. For example, the elPrep [27] tool can be used as a drop-in replacement for certain modules of the GATK software suite. Alternatively, certain workflows such as ADAM/avocado [28] and certain GATK modules provide variant calling pipelines that are implemented in native Spark itself without relying on existing tools. However, such an approach is rarely used because it requires extensive programming efforts.

In contrast to existing tools that focus on germline variant calling using either DNA-sequencing or RNA-sequencing data, we focus in this work on somatic variant calling from DNA-sequencing data. To the best of our knowledge, Halvade Somatic is the first software framework to leverage a Big Data processing platform for this task.

## Implementation

### Apache Spark

Apache Spark is a data processing framework that was built to overcome some of the limitations of Hadoop MapReduce. Both frameworks share common principles such as the use of a distributed file system to provide scalable access to large volumes of data and support for parallel data processing in a fault-tolerant manner. Compared to MapReduce, Apache Spark allows for a wider range of operations through its API implemented in several programming languages. Additionally, Spark avoids disk I/O when possible: data are kept in memory for as long as possible and only written to disk when the data volume exceeds the memory capacity or when explicitly asked to persist data on disk. We briefly describe the most important terminology of Spark. For a more detailed account, we refer to [11].

Data is stored in Spark using “Resilient Distributed Datasets” (RDDs). RDDs can be thought of as containers for large volumes of data that are partitioned into smaller chunks that are distributed over the local memories of the worker nodes. Operations on data are performed through “transformations” and “actions.” Transformations apply a particular operation to an RDD, yielding a new RDD that is again distributed over the worker nodes. In contrast, actions on RDDs apply operations for which the result is collected in the driver program.

Spark relies on “lazy evaluation” of RDDs. Subsequent transformations on RDDs form the Spark “lineage,” which is evaluated only when an action is triggered. This is called a Spark “job.” The actual computations are performed by “executors,” i.e., processes on the worker nodes that are in charge of running individual tasks on subsets of the data. Lazy evaluation allows performance to be optimized because certain operations can be grouped together. Fault tolerance is provided by recomputing lost results: if an executor fails, the lineage is used to recalculate results starting from the last available data. When the data of an RDD are used multiple times and/or when the computation of an RDD is costly, it is beneficial to “persist” the RDD, which means that its data are explicitly stored in memory or on disk.

To execute existing tools (e.g., BWA or GATK) inside the Spark framework, we created a specialized PipedRDD implementation that supports common bioinformatics formats such as SAM or BAM [29]. SAM records are represented as an iterator over strings while BAM data are represented as an array of bytes.

### Halvade Somatic

**Figure 2:**
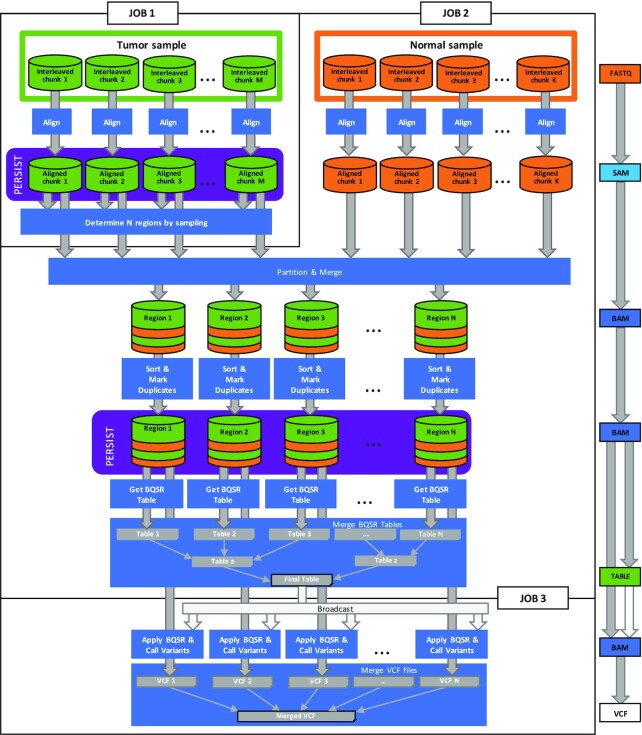
: Overview of the somatic variant calling framework in Spark. The workflow consists of 3 Spark jobs where the data at the end of Jobs 1 and 2 are persisted. During the first job, the reads of the tumor sample are aligned to the reference genome and *N* chromosomal regions are determined such that each region contains roughly an equal number of aligned tumor reads. In the second job, the reads of the normal sample are aligned. The aligned reads (tumor and normal) are grouped per chromosomal region. Next, for each genomic region independently, reads are sorted according to the position to which they align and read duplicates are marked. This output is again persisted. Per genomic region, partial BQSR statistics are computed and merged into a genome-wide table. The last job uses this merged table to apply the BQSR to each read and call the somatic variants in all regions. The variants are merged into a single VCF output file. Note that certain tools in the workflow also require the (indexed) reference genome or dbSNP database. For simplicity, these input files are not shown.

Halvade Somatic leverages Apache Spark for the parallel, distributed-memory processing of the somatic variant calling pipeline shown in Fig. 1. A global overview of Halvade Somatic is depicted in Fig. 2. The workflow consists of 3 Spark jobs.

In the first job, the reads of the tumor DNA sample are aligned against the reference genome. Next, the reference genome is partitioned into *N* chromosomal regions in such a way that the regions contain roughly an equal number of aligned reads. To have an accurate, yet computationally efficient algorithm to determine the region boundaries, this procedure is performed on a randomly sampled subset of the aligned reads.

During the second job, the reads of the matched normal sample are aligned against the reference genome. The aligned read records of the tumor and normal samples are grouped according to the *N* regions that were established during the first job. Next, for each of the *N* regions independently, reads are sorted according to the position to which they align, read duplicates are marked, and BQSR statistics are computed. These *N* partial BQSR statistics are aggregated into a single, genome-wide BQSR table.

Finally, in the third job, BQSR is applied to all reads. Somatic variants are called for each region independently. The *N* resulting partial Variant Call Format (VCF) files are merged into a single VCF output file.

Below, the different computational steps are described in more detail.

#### Input data preparation

Halvade Somatic supports input data as either unaligned reads (in FASTQ format) or pre-aligned reads (in BAM format). In the former case, paired-end reads, per read group, are typically provided as 2 distinct, compressed (gzipped) FASTQ files. Halvade Somatic decompresses these files and splits them into smaller chunks (default size: 60 MB) that are distributed across worker nodes in such a way that paired-end reads are kept together. These chunks later serve as input for the alignment tasks that are executed in Jobs 1 and 2 for the tumor and normal sample, respectively.

For performance reasons, the process of splitting data into chunks is multi-threaded. In case the data are provided as multiple read groups (and hence, multiple pairs of FASTQ files), this preprocessing step is performed by multiple Spark executors (i.e., multiple processes that are executed in parallel), 1 executor per read group. Often, the runtime of this preprocessing step is governed by data I/O.

Alternatively, the input data can be provided as pre-aligned BAM files. These BAM files are stored on the associated distributed file system (e.g., HDFS or Amazon S3) and parsed efficiently using Hadoop-BAM [30].

#### Read alignment, partitioning, and merging

Assuming unaligned input, multiple Spark executors run, in parallel, an instance of BWA [7] to align the reads of the tumor sample against the reference genome. Each BWA instance reads a FASTQ chunk from disk and streams the aligned SAM records to a PipedRDD. Because the total number of FASTQ chunks is typically much higher than the number of executors, each executor has to process several chunks. Spark assigns chunks to executors such that the workload is evenly balanced while taking into account data locality. In case multiple CPU cores are assigned per executor, the multi-threading functionality of BWA is used. The resulting RDD that holds the aligned SAM records is persisted because it is a dependency for multiple later steps. Because this RDD contains several hundreds of GB of data, it is persisted to disk by default.

Next, the reference genome is partitioned into *N* non-overlapping chromosomal regions. At a later stage, the preprocessing and variant calling steps will be parallelized by these regions. The value of *N* is user-defined (default: 1,800) and is typically much higher than the number of executors. The size of the chromosomal regions is non-uniform and is determined such that each region contains roughly the same number of aligned (tumor) reads. By accounting for possible variance in coverage among regions, we avoid regions with an excessive number of aligned reads and we obtain better load balancing compared to using uniformly sized regions. For efficiency reasons, only a relatively small, randomly sampled subset of the tumor reads (default: 60*N* reads) is used to determine the size of the chromosomal regions. This action concludes the first Spark job.

In the second Spark job, the reads of the normal sample are aligned to the reference genome. The RDDs that contain the aligned tumor and normal reads are merged and partitioned according to the previously determined *N* chromosomal regions. This task requires the shuffling of large volumes of aligned read records and hence relies on inter-node communication. Read pairs that span the boundary of adjacent regions are duplicated in both regions.

#### Sorting, marking read duplicates, BQSR, and variant calling

After partitioning into regions, the reads are further sorted according to the chromosomal position to which they align. Data are spilled to disk if insufficient RAM is available, similar to how SAMtools [29] sorts SAM records. Sorted reads are written to BAM files on disk, 1 BAM file per chromosomal region.

PCR and optical read duplicates cannot be considered as independent observations during variant calling and should therefore be marked accordingly. To this end, per chromosomal region independently, instances of the GATK “Mark Duplicates (Picard)” module are run. The resulting RDD is again persisted to disk. Our PipedRDD implementation supports running multiple instances of GATK per executor. This yields a significant performance increase when multiple CPU cores are assigned per executor and when the tool does not efficiently support multi-threading. Because GATK shows an average CPU usage of 100–200%, we assign 1 GATK instance per 2 CPU cores.

BQSR corrects for systematic errors when the sequencing machine estimates per-base quality scores. Per chromosomal region independently, a BQSR table is constructed using the “BaseRecalibrator” module of GATK. A BQSR table summarizes empirically observed information on the quality score distribution and is required for the actual recalibration step. We avoid counting reads that span region boundaries (and that are present in both regions) twice.

Because the accuracy of the BQSR depends on the volume of the observed data and owing to variability among the chromosomal regions we choose to aggregate these partial tables into a single *genome-wide* table using the TreeReduce action in Spark, concluding the second job. Even though this process requires inter-process communication and hence the synchronization of the different subtasks, we observed that merging the partial BQSR tables is essential to have a good correspondence between the variants called by the original (sequential) pipeline and those called by Halvade Somatic.

In the third Spark job, the merged BQSR table is distributed to all executors and the “ApplyBQSR” module of GATK is executed. Finally, somatic variants are called using either Mutect2 or Strelka2, per chromosomal region independently, thus producing 1 VCF file per chromosomal region that is stored using the Spark saveAsTextFile action. These partial VCF files are merged into a single VCF output file.

Optionally, if somatic variants from both Mutect2 and Strelka2 are desired, the BAM file that resulted from the BQSR step is persisted in order to avoid its recomputation. The second somatic variant caller is then run as a fourth Spark job (not shown in Fig. 2).

#### Spark configuration

The correct configuration of the number of executors per worker node is essential for good performance. Note that the number of executors per node remains fixed across the different Spark jobs. In principle, a high number of executors is preferred to maximize parallelism in Spark. However, owing to limited hardware resources, the number of executors is often restricted. When read mapping is required (FASTQ input), an executor requires ∼16 GB RAM (8 GB for the BWA instance, 6 GB for the executor, and 2 GB for executor overhead). This constraint often limits the number of executors. For example, for worker nodes with 64 GB of RAM, this translates into 4 executors per worker node. When the alignment step is not required (BAM input), the memory per executor can be reduced to ∼1 GB per GATK instance, 6 GB for the executor, and 2 GB overhead. The availability of more memory can improve performance in Spark because it reduces the chance of having to spill data to disk. The available CPU cores are evenly assigned to the different executors. We run multiple instances of a tool in parallel per executor if enough CPU cores are available. With this we can effectively increase CPU utilization and decrease overall runtime.

A second performance-critical parameter is the number of chromosomal regions *N*. More (and hence: smaller) regions lead to reduced memory requirements per executor but a higher tool starting overhead (e.g., GATK tries to check whether it is running on a Google Cloud node, which can take several seconds). Additionally, a higher value of *N* increases the number of reads that need to be duplicated across adjacent regions. Nevertheless, using only a few regions increases the volume of data per region, leading to increased memory requirements and difficulties in evenly balancing the workload. From tests, we conclude that using 1,500*n*–1,800*n* regions is optimal for a typical WGS sample and 250*n*–320*n* for a typical WES sample. Here, *n* denotes the number of GATK instances per executor.

## Results

### Data and Availability

All WGS benchmarks were performed using 100-bp, paired-end Illumina HiSeq 2000 reads of a breast cancer sample (HCC1395) with a matched normal lymphoblastoid cell line (HCC1395 BL). Data are available through the Genome Modeling System [31,32] project and consist of ∼1 billion reads (normal sample) and 1.88 billion reads (tumor sample), translating to sequencing depths of 34× and 62×, respectively.

WES benchmarks were performed using 100-bp, paired-end Illumina reads of the TCGA-A8-A08F sample. Data are available through the Cancer Genome Atlas Breast Invasive Carcinoma (TCGA-BRCA) data collection. The tumor sequencing data consist of 201 million reads, while the blood-derived normal sequencing data consist of ∼156 million reads.

The Genome Reference Consortium Human build 38 (GRCh38) reference was used.

### Performance Benchmarks

We first assess the computational performance of Halvade Somatic on a private computer cluster with 36 CPU cores (dual 2.30-GHz Intel® Xeon® Gold 6140 CPUs) and 187 GB of RAM per node. The worker nodes are connected to a General Parallel File System (GPFS) with a high-performance Enhanced Data Rate (EDR) Infiniband network. We used Spark 3.0.0, Hadoop Yarn 2.9.2, BWA 0.7.16a, Samtools 1.5, GATK 4.1.2.0, and Strelka 2.9.10. Halvade Somatic further relies on the HadoopBAM 7.10.0 and HtsJDK 2.11.0 libraries.

When input is provided as unaligned reads (FASTQ files), we use 9 executors per worker node, except for the worker node that also runs the Spark driver program, which has 8 executors. Each executor is thus allocated 4 CPU cores and ∼20 GB of memory. A single instance of BWA with 4 threads is run per executor while each executor runs 2 instances of GATK. When input is provided as aligned reads (BAM files), we use 18 executors per node (17 for the node that runs the driver), with 2 CPU cores and ∼10 GB per executor. In that case, a single instance of GATK per executor is run.

**Figure 3:**
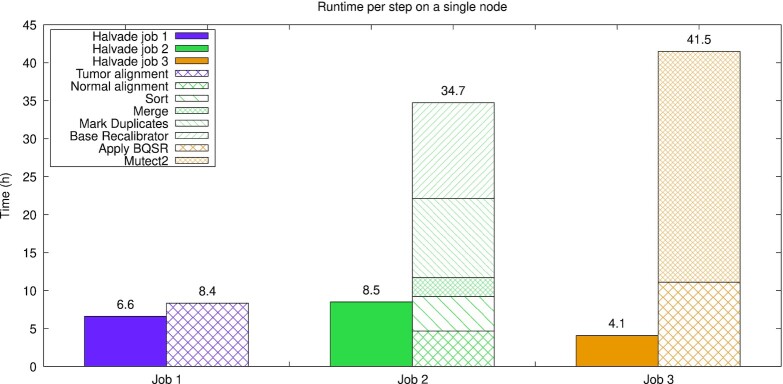
: Comparison and breakdown of the runtime of Halvade Somatic and the original Mutect2 pipeline on a single node. Owing to efficient multi-threading support in BWA, the reduction in runtime for Job 1 is limited. Jobs 2 and 3 show a significant reduction in runtime due to the limited support for multi-core architectures in GATK/Mutect2.

Table 1 shows the runtimes of the original pipeline and Halvade Somatic for different combinations of input (FASTQ or BAM), different samples (WGS or WES), and somatic variant calling tools (Mutect2, Strelka2, or both). The original pipeline can be run only on a single node and multi-threading was enabled for all tools that support it. Even on a single node, Halvade Somatic considerably reduces the runtime: when Mutect2 is used as a somatic variant calling tool, runtime is reduced from 84.57 to 19.45 h, a speedup of 4.34 times. Figure 3 shows a detailed breakdown of the runtime over the different steps. Clearly, the largest gains are obtained during Spark Jobs 2 and 3, owing to the under-utilization of hardware resources by GATK and Mutect2. Even though BWA has efficient support for multi-threading, Halvade Somatic is able to slightly reduce the runtime of alignment steps as well.

**Table 1.** Runtime of the original pipeline and Halvade Somatic for different combinations of samples (WGS or WES), input (FASTQ or BAM), and somatic variant calling tools (Mutect2, Strelka2, or both).

Input	Variant caller	Original pipeline (h)	Halvade Somatic (h)					
		1 node	1 node	2 nodes	4 nodes	8 nodes	12 nodes	16 nodes
WGS								
FASTQ	Mutect2	84.57	19.45	9.35	4.81	2.47	1.74	1.36
FASTQ	Strelka2	55.66	18.74	9.19	4.45	2.31	1.57	1.21
FASTQ	Both	86.03	21.89	10.50	5.22	2.74	1.90	1.51
BAM	Mutect2	71.53	10.09	5.28	2.47	1.21	0.94	0.73
BAM	Strelka2	42.62	9.94	5.24	2.28	1.07	0.83	0.61
BAM	Both	72.99	12.77	6.91	2.99	1.53	1.13	0.96
WES								
FASTQ	Mutect2	12.59	2.38	1.21				
FASTQ	Strelka2	7.03	1.63	0.82				
FASTQ	Both	12.66	2.65	1.36				
BAM	Mutect2	10.72	1.70	0.85				
BAM	Strelka2	5.16	0.86	0.42				
BAM	Both	10.79	1.90	1.04				

Strelka2 is considerably faster than Mutect2 and has efficient support for multi-threading. However, using a single node, Halvade Somatic is still 2.97 times faster (55.66 versus 18.74 h, see Table 1) for the entire pipeline. Running both Mutect2 and Strelka2 requires only a little extra runtime compared to running only Mutect2. Hence, the use of both variant callers appears attractive to create a high-confidence set of somatic variants as also proposed in the literature [17,33].

When pre-aligned (BAM) input is provided, the alignment step can be omitted and runtime decreases accordingly. The relative gain from using Halvade Somatic is even more pronounced, as in this case, the pipeline predominantly consists of the GATK and Mutect2 steps. For example, when running both variant callers on a single node on the WGS dataset, the runtime is reduced from 72.99 to 12.77  h (see Table 1), a speedup of 5.72 times. Similarly, when using WES data, we observe, depending on input type and variant caller, speedups ranging from 4.32 to 6.31 times.

**Figure 4:**
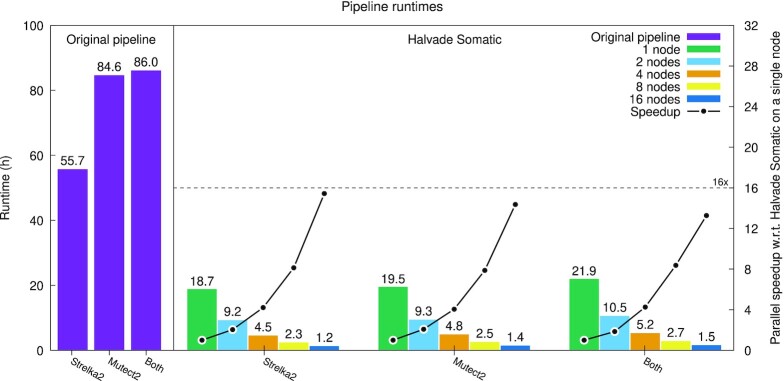
: Runtime and parallel speedup for the WGS sample using FASTQ input.

For time-critical samples, Halvade Somatic can further reduce runtime by scaling to multiple worker nodes. Figure 4 shows the parallel speedup obtained for the WGS sample and the different variant calling tools. The parallel speedup *S_p_* is the ratio of runtime using a single node *T*_1_ and the runtime using *p* nodes *T_p_*. In the ideal case, *S_p_* equals the number of nodes *p*. Owing to communication and synchronization overhead, the observed speedups are slightly lower. Using 16 nodes and FASTQ input, we observe an additional parallel speedup that ranges between 14.4 times (Mutect2 pipeline) and 15.4 times (Strelka2 pipeline). This translates into a high parallel efficiency η_*p*_ = *S_p_*/*p* of, respectively, 89.7% and 96.3%, indicating that Halvade Somatic efficiently uses the extra hardware resources to reduce runtime. The value 1/η_*p*_ − 1 (respectively, 11.4% and 3.8%) expresses the additional cost (e.g., financial or energy) of a multi-node run relative to single-node execution.

The combined effect of improved resource utilization of a node and the use of multiple nodes is significant: using the Mutect2 pipeline, the WGS sample, and FASTQ input, runtime is reduced from 84.57 h (original pipeline, single node) to 1.36 h (Halvade Somatic, 16 nodes), an overall speedup of 62.4 times. Similarly, using the Strelka2 variant caller, runtime is reduced from 55.66 h (original pipeline, single node) to 1.21 h (Halvade Somatic, 16 nodes).

### Cloud and Docker support

#### Docker image

We provide a Docker image to facilitate the deployment of Halvade Somatic on a node without native Spark installation. The image contains all necessary software packages and libraries.

The use of a Docker image imposes virtually no computational overhead: using a node with 32 CPU cores (dual 2.30 GHz Intel® Xeon® CPU E5-2698 v3) and 256 GB of memory, we measured a runtime for the WGS sample of 20.59 and 9.55 h for FASTQ and BAM input, respectively. For the WES sample we measured a runtime of 2.10 and 1.25 h for FASTQ and BAM input, respectively.

#### Amazon EMR

Halvade Somatic can also be deployed on public cloud compute platforms such as Amazon EMR. Input data, reference files, binaries, and libraries should be uploaded to Amazon S3 storage. We provide a bootstrap script to copy certain files from Amazon S3 storage to the individual worker nodes, a task that requires ∼10 minutes. We benchmarked Halvade Somatic using an r5d.xlarge node (2 CPU cores, 32 GB of RAM, and a single 150-GB NVMe SSD) to run the driver program and r5d.8xlarge nodes (16 CPU cores, 256 GB of RAM, and 2 × 600 GB NVMe SSDs) as worker nodes. The runtime of Halvade Somatic for the Mutect2 pipeline is reported in Table 2 for the different samples, input type, and a different number of nodes, along with the total financial cost using standard Amazon pricing.

**Table 2.** Runtime of Halvade somatic for the Mutect2 pipeline on Amazon EMR

Input	No. of nodes	Halvade Somatic runtime (h)	Cost (USD)
WGS			
FASTQ	8	3.25	83.81
BAM	8	1.43	41.90
WES			
FASTQ	1	2.75	8.80
FASTQ	2	1.42	11.02
BAM	1	1.88	5.87
BAM	2	1.08	11.02

The cost is calculated using standard pricing of region eu-west-1 (Ireland) at the time of writing.

### Assessment of variant accuracy

The resulting VCF file can differ slightly between a parallelized Halvade Somatic run and the corresponding sequential pipeline. We emphasize that the set of somatic variants called by the original pipeline most likely does not fully correspond to the biological ground truth and is hindered by false-positive and false-negative variant calls. It is well known that somatic variant calling is a notoriously difficult problem and different somatic variant calling tools often show limited overlap in their output (see, e.g., [34]). In this section, we pinpoint the origins of the small differences that arise purely as a result of the parallelization of the pipeline itself.

**Figure 5:**
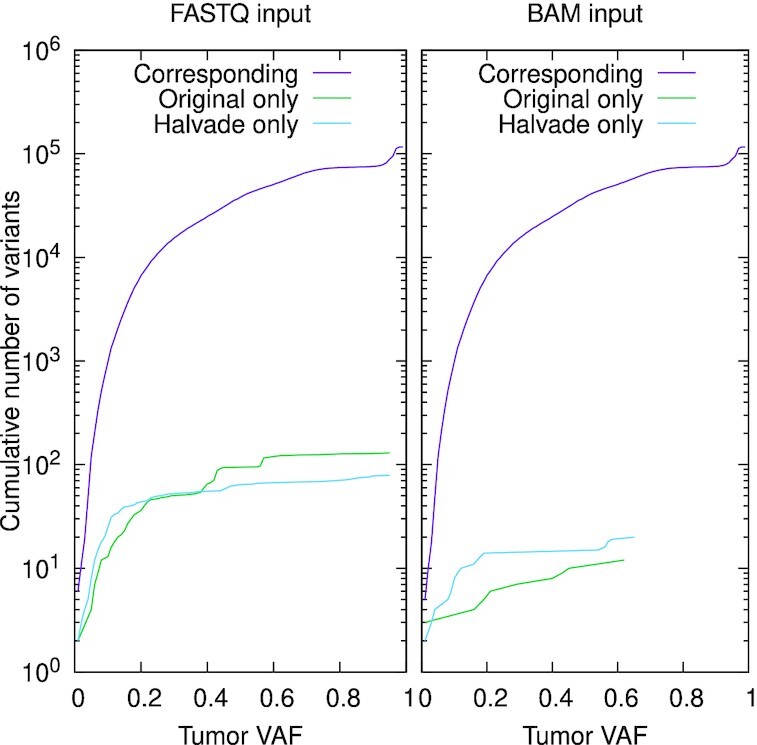
: Cumulative number of corresponding and discordant somatic variants between the original, sequential pipeline and Halvade Somatic as a function of the tumor variant allele frequency (VAF) for FASTQ input (left) and BAM input (right). “Corresponding” refers to somatic variants identified by both methods; “Original only” refers to somatic variants called only by the original, sequential pipeline; “Halvade only” refers to somatic variants identified only by Halvade Somatic. In all cases, the Mutect2 variant caller was used.

Using the original, sequential GATK/Mutect2 pipeline and WGS data, we find 116, 791 somatic variants after filtering with the GATK “FilterMutectCalls” module. Starting from FASTQ input, Halvade Somatic identifies 116 ,661 overlapping (99.89%), 130 missed (0.11%), and 79 additional (0.07%) somatic variants (see Fig. 5). Most of these differences are due to differential read alignment: because of parallelization, the order in which (paired-end) reads are presented to BWA causes output differences. This is due to the random placement of repetitive reads and the fact that the fragment size of paired-end reads may be estimated slightly differently for different FASTQ chunks. To confirm this, we ran the original GATK/Mutect2 pipeline on a shuffled FASTQ file and observed the same degree of variability in resulting somatic variants (data not shown).

When using Halvade Somatic with pre-aligned BAM input, we eliminate this source of variation and identify 116, 779 overlapping (99.99%), 12 missed (0.01%), and 20 additional (0.017%) somatic variants. These small differences in output are caused by subtle variability during the mark duplicates step that may occur for reads that span region boundaries. Additionally, Mutect2 uses random downsampling at positions with extremely high coverage.

We conclude that the variants called by Halvade Somatic match those of the original pipeline to a very high degree and that very small differences in output are mostly due to random effects.

## Discussion and Conclusion

The accurate identification of somatic variants from NGS data is time consuming, especially when WGS data are used. Individual tools for read mapping, data preparation, and variant calling have matured but often lack support for multi-node and sometimes even multi-core computer systems. This, in turn, translates to high execution times—often days—to process raw sequencing data. For germline variant calling several software tools have been proposed in the literature that leverage Big Data platforms such as MapReduce or Spark to strongly reduce runtime. For the problem of somatic variant calling, however, such tools are lacking.

Halvade Somatic implements the somatic variant calling pipeline according to the GATK best practices recommendations. It supports both the Mutect2 and Strelka2 variant callers and takes advantage of the Apache Spark framework to call somatic variants with high computational performance, scalability, and reliability. Most of the workload can be parallelized: reads can be mapped in parallel, while data preprocessing steps and variant calling can be parallelized by genomic region. Spark is used to create and manage parallel data streams and run multiple instances of tools in parallel on subsets of the data. To partition and sort aligned SAM records, and to build a genome-wide BQSR table, we rely on Spark communication primitives to exchange the relevant data among worker nodes.

Halvade Somatic drastically reduces runtimes even on a single node: depending on the exact set-up (WES or WGS, FASTQ or BAM input, choice of variant caller), we measured a speedup ranging from 2.97 to 6.31 times. Halvade also scales well across multiple nodes if a larger cluster is available. We observe parallel speedups of 13.27 times and higher when scaling to 16 nodes.

Extensive documentation is available online (https://halvadeforspark.readthedocs.io). A Docker image is provided to run Halvade on a single node. Cloud support is available through Amazon EMR and the Google Cloud.

## Availability of Source Code and Requirements

- Project name: Halvade Somatic
- Project home page: https://bitbucket.org/dries_decap/halvadeforspark/src/master/
- Operating system(s): Linux
- Programming language: Scala
- Other requirements: Apache Spark 3.0 or higher, GATK 4.1.2.0 or higher, Samtools 1.5 or higher, and BWA 0.7.16 or higher
- biotoolsID: halvade_somatic
- RRID: SCR_021771
- License: GPL v3.0

## Data Availability

The human genome reference GRCh38 and all required reference files used in this article are publicly available through the Resource bundle of GATK at https://console.cloud.google.com/storage/browser/genomics-public-data/resources/broad/hg38/v0/. We used the Homo_sapiens_assembly38.fasta reference file and Homo_sapiens_assembly38.known_indels.vcf.gz, which contains the known variants. The HCC1395 WGS sample [31] used in all benchmarks in this article is publicly available as well at http://genomedata.org/pmbio-workshop/fastqs/all/. The WES sample are available through the TCGA-BRCA data collection at https://portal.gdc.cancer.gov/cases/0a017f15-1c6b-45e7-8d55-e0a71df1b2e8. Detailed documentation to run and use Halvade is available at https://halvadeforspark.readthedocs.io/en/latest. Snapshots of our code and other data further supporting this work are openly available in the GigaScience repository, GigaDB [35].

## Abbreviations

API: Application Programming Interface; BAM: Binary Sequence Alignment/Map; bp: base pairs; BQSR: Base Quality Score Recalibration; BWA: Burrows-Wheeler Aligner; CPU: central processing unit; EDR: enhanced data rate; GATK: Genome Analysis ToolKit; GPFS: General Parallel File System; HCC: Human Cancer Cell Line; HDFS: Hadoop Distributed File System; GRCh38: Genome Reference Consortium Human build 38; NGS: next-generation sequencing; RAM: random-access memory; RDD: Resilient Distributed Datasets; SAM: Sequence Alignment/Map Format; TCGA: The Cancer Genome Atlas Program; TCGA-BRCA: Cancer Genome Atlas Breast Invasive Carcinoma; VAF: variant allele frequency; VCF: Variant Call Format; WGS: whole-genome sequencing; WES: whole-exome sequencing.

## Competing Interests

The authors declare that they have no competing interests.

## Funding

This research is conducted within the project entitled “ATHENA – Augmenting Therapeutic Effectiveness through Novel Analytics,” project No. HBC.2019.2528, funded by VLAIO (Flanders Innovation & Entrepreneurship).

## Authors' Contributions

D.D. designed and developed Halvade Somatic. P.C., C.H., and R.W. assisted with the performance analysis. L.S.B., M.L., and K.M. aided with the accuracy assessment. J.F. supervised the work. D.D. and J.F. wrote the manuscript. All authors read and approved the manuscript.

## Supplementary Material

giab094_GIGA-D-21-00266_Original_Submission

giab094_GIGA-D-21-00266_Revision_1

giab094_GIGA-D-21-00266_Revision_2

giab094_Response_to_Reviewer_Comments_Original_Submission

giab094_Reviewer_1_Report_Original_Submission Medhat Mahmoud -- 9/27/2021 Reviewed

giab094_Reviewer_1_Report_Revision_1 Medhat Mahmoud -- 11/7/2021 Reviewed

giab094_Reviewer_2_Report_Original_Submission Jiarui Ding, Ph.D. -- 10/2/2021 Reviewed

giab094_Reviewer_2_Report_Revision_1 Jiarui Ding, Ph.D. -- 11/2/2021 Reviewed

giab094_Reviewer_3_Report_Original_Submission Zaid Al-Ars -- 10/16/2021 Reviewed
