# Supplementary material for: Halvade somatic: Somatic variant calling with Apache Spark
Source: Gigascience. 2022 Jan 12;11:giab094. doi: 10.1093/gigascience/giab094 (PMC8756192; doi:10.1093/gigascience/giab094)
Supplement: giab094_GIGA-D-21-00266_Original_Submission [file giab094_giga-d-21-00266_original_submission.pdf]

GigaScience

Halvade Somatic: Somatic Variant Calling with Apache Spark

--Manuscript Draft--

**Manuscript Number:**	GIGA-D-21-00266
**Full Title:**	Halvade Somatic: Somatic Variant Calling with Apache Spark
**Article Type:**	Technical Note
**Funding Information:**	Agentschap Innoveren en Ondernemen (HBC.2019.2528) — Prof. Jan Fostier
**Abstract:**	Background: The accurate detection of somatic variants from sequencing data is of key importance for cancer treatment and research. Somatic variant calling requires a high sequencing depth of the tumor sample, especially when also the detection of low-frequency variants is desired. In turn, this leads to large volumes of raw sequencing data to process and hence, large computational requirements. For example, calling the somatic variants according to the GATK best practices guidelines requires days of computing time for a typical whole genome sequencing sample.<br><br>Findings: We introduce Halvade Somatic, a framework for somatic variant calling from DNA sequencing data that takes advantage of multi-node and/or multi-core compute platforms to reduce runtime. It relies on Apache Spark to provide scalable I/O and to create and manage data streams that are processed on different CPU cores in parallel. Halvade Somatic contains all required steps to process the tumor and matched normal sample according to the GATK best practices recommendations: read alignment (BWA), sorting of reads, preprocessing steps such as marking duplicate reads and base quality score recalibration (GATK) and finally, calling the somatic variants (Mutect2). Our approach reduces the runtime on a single 36-core node to 19.5h compared to a runtime of 84.5h for the original pipeline, a speedup of 4.3x. Runtime can be further decreased by scaling to multiple nodes while retaining a high parallel efficiency, e.g., we observe a runtime of 1h 21min using 16 nodes, an additional speedup of 14.4x. The somatic variants called by Halvade Somatic match those obtained by the original GATK/Mutect2 pipeline to a very high degree. Halvade Somatic supports variant calling from both whole genome sequencing (WGS) as well as whole exome sequencing (WES) data and also supports Strelka2 as an alternative or complementary variant calling tool within its pipeline. We provide a Docker image to facilitate single node deployment. Halvade Somatic can be executed on a wide variety of compute platforms, including the Amazon EC2.<br><br>Conclusions: Halvade Somatic is the first somatic variant calling pipeline that leverages big data processing platforms and provides reliable, scalable performance. Source code is freely available.
**Corresponding Author:**	Jan Fostier<br>Ghent University: Universiteit Gent<br>Ghent, BELGIUM
**Corresponding Author Secondary Information:**	
**Corresponding Author's Institution:**	Ghent University: Universiteit Gent
**Corresponding Author's Secondary Institution:**	
**First Author:**	Dries Decap
**First Author Secondary Information:**	
**Order of Authors:**	Dries Decap
	Louise de Schaetzen van Brienen
	Maarten Larmuseau
	Pascal Costanza

Powered by Editorial Manager® and ProduXion Manager® from Aries Systems Corporation

	Charlotte Herzeel
	Roel Wuyts
	Kathleen Marchal
	Jan Fostier
**Order of Authors Secondary Information:**	
**Additional Information:**	
**Question**	**Response**
Are you submitting this manuscript to a special series or article collection?	No
**Experimental design and statistics**<br><br>Full details of the experimental design and statistical methods used should be given in the Methods section, as detailed in our Minimum Standards Reporting Checklist. Information essential to interpreting the data presented should be made available in the figure legends.<br><br>Have you included all the information requested in your manuscript?	Yes
**Resources**<br><br>A description of all resources used, including antibodies, cell lines, animals and software tools, with enough information to allow them to be uniquely identified, should be included in the Methods section. Authors are strongly encouraged to cite Research Resource Identifiers (RRIDs) for antibodies, model organisms and tools, where possible.<br><br>Have you included the information requested as detailed in our Minimum Standards Reporting Checklist?	Yes
**Availability of data and materials**<br><br>All datasets and code on which the conclusions of the paper rely must be either included in your submission or	Yes

deposited in publicly available repositories (where available and ethically appropriate), referencing such data using a unique identifier in the references and in the “Availability of Data and Materials” section of your manuscript.<br><br>Have you have met the above requirement as detailed in our Minimum Standards Reporting Checklist?	

Manuscript

Click here to access/download;Manuscript;Somatic_Halvade_GigaScience.

GigaScience, 2021, 1–16

doi: xx.xxxx/xxxx

Manuscript in Preparation

Technical Note

TECHNICAL NOTE

Halvade Somatic: Somatic Variant Calling with Apache Spark

Dries Decap[1], Louise de Schaetzen van Brienen[1], Maarten Larmuseau[1], Pascal Costanza[2], Charlotte Herzeel[3], Roel Wuyts[3], Kathleen Marchal[1] and Jan Fostier[1,*]

[1]IDLab, Ghent University - imec, Technologiepark 126, B-9052 Ghent, Belgium and [2]Intel, Belgium and [3]imec, Kapeldreef 75, B-3001 Leuven, Belgium

[*]jan.fostier@ugent.be

Abstract

Background: The accurate detection of somatic variants from sequencing data is of key importance for cancer treatment and research. Somatic variant calling requires a high sequencing depth of the tumor sample, especially when also the detection of low-frequency variants is desired. In turn, this leads to large volumes of raw sequencing data to process and hence, large computational requirements. For example, calling the somatic variants according to the GATK best practices guidelines requires days of computing time for a typical whole genome sequencing sample.

Findings: We introduce Halvade Somatic, a framework for somatic variant calling from DNA sequencing data that takes advantage of multi-node and/or multi-core compute platforms to reduce runtime. It relies on Apache Spark to provide scalable I/O and to create and manage data streams that are processed on different CPU cores in parallel. Halvade Somatic contains all required steps to process the tumor and matched normal sample according to the GATK best practices recommendations: read alignment (BWA), sorting of reads, preprocessing steps such as marking duplicate reads and base quality score recalibration (GATK) and finally, calling the somatic variants (Mutect2). Our approach reduces the runtime on a single 36-core node to 19.5 h compared to a runtime of 84.5 h for the original pipeline, a speedup of 4.3 ×. Runtime can be further decreased by scaling to multiple nodes while retaining a high parallel

Key Points

- Typically, somatic variant calling involves sequencing a tumor sample and matched normal sample. To capture also low-frequency variants, a high sequencing depth of the tumor sample is warranted. Hence, large volumes of raw sequencing data must be processed, a task that requires significant computational resources, especially for whole genome sequencing.
- Halvade Somatic is the first pipeline to leverage Big Data platforms for somatic variant calling and is based on the GATK/Mutect2 pipeline. Using Apache Spark, we provide a scalable and reliable pipeline that strongly reduces runtime. For example, runtime is reduced from 84.5 h (single 36-core node with original pipeline) to 1 h 21 min (a cluster of 16 nodes with Halvade Somatic), a speedup of 62.4 ×.
- We provide the ability to use Strelka2 as an alternative, or complementary variant calling tool. In the latter case, two variant callsets are generated which can be combined and filtered to generate high-confidence consensus variants.
- Both whole genome sequencing and whole exome sequencing data are supported.
- Variants produced by Halvade Somatic match those of the original GATK/Mutect2 pipeline to a very high degree: 99.89% when starting from raw sequencing data (FASTQ) and 99.99% when staring from pre-aligned BAM input.
- All code is publicly available under the GPL v3.0 license. A Docker container is provided to facilitate single-node deployment without local Spark installation.

efficiency, e.g., we observe a runtime of 1 h 21 min using 16 nodes, an additional speedup of 14.4 ×. The somatic variants called by Halvade Somatic match those obtained by the original GATK/Mutect2 pipeline to a very high degree. Halvade Somatic supports variant calling from both whole genome sequencing (WGS) as well as whole exome sequencing (WES) data and also supports Strelka2 as an alternative or complementary variant calling tool within its pipeline. We provide a Docker image to facilitate single node deployment. Halvade Somatic can be executed on a wide variety of compute platforms, including the Amazon EC2.

Conclusions: Halvade Somatic is the first somatic variant calling pipeline that leverages big data processing platforms and provides reliable, scalable performance. Source code is freely available.

Key words: Apache Spark; Somatic Variant Calling; GATK/Mutect2; Strelka2

Somatic mutations are changes in the DNA of a cell that are introduced during the lifetime of a living organism. Due to their role in the development of cancer, the accurate detection of somatic variants is of key importance. The broad landscape of somatic variants has been characterized by large-scale research projects such as The Cancer Genome Atlas Program (TCGA) [1], The Cancer Cell Line Encyclopedia (CCLE) [2] and the International Cancer Genome Consortium (ICGC) [3]. In clinical practice, the profiling of genomic variants and signatures in tumors is increasingly adopted to provide patient-tailored therapies.

Cancer mutations are often characterized using next-generation sequencing (NGS) technology. In a typical setting, a tumor sample is accompanied by a matched normal sample from which germline variants are determined. Cancer-specific mutations are those that are present in the tumor sample but absent from the normal sample. The tumor sample is often heterogeneous: it may contain different subpopulations of cancer cells with distinct molecular signatures [4]. As such, mutations can appear in a bulk tumor sample with varying frequency. In order to capture also low-frequency variants, a high sequencing depth of the tumor sample is warranted (typically $\geq 50\times$) [5]. Together with the sequencing of the matched normal, this gives rise to large volumes of raw sequencing data, especially for whole genome sequencing (WGS). In turn, this leads to high processing times. To illustrate this, we consider the variant calling pipeline according to the GATK best practices recommendations [6] that uses BWA [7] for read mapping, Picard [8] and GATK [9] for data preprocessing and Mutect2 [10] for somatic variant calling. To process

Compiled on: September 1, 2021.
Draft manuscript prepared by the author.

an Illumina HiSeq 2000 whole genome sequencing dataset of the HCC1395 sample (breast cancer cell line) with a sequencing depth of 62× (tumor) and 34× (normal) and using a 36-core machine (dual 2.30 GHz Intel Xeon Gold 6140 CPU with 196 GB of RAM), we measured a runtime of roughly three and a half days (~84.5 h): ~13 h for read mapping, ~41 h for data preprocessing and ~30.5 h for variant calling. This very high runtime is caused not only by the large volume of input sequencing data to process (~693 GiB uncompressed) but also due to the fact that Picard, GATK and Mutect2 do not efficiently make use of modern, multi-core architectures as most of their codebase is single-threaded. As such, the computational resources provided by modern compute systems are underutilized.

We present Halvade Somatic, a scalable software framework that leverages Apache Spark [11] to efficiently perform somatic variant calling using multi-node and/or multi-core compute platforms. Halvade Somatic creates and manages parallel data streams that are processed by multiple instances of existing tools on different CPU cores. It implements the somatic variant calling pipeline according to the GATK best practices recommendations (see Fig. 1 for an overview). Next to Mutect2 [10], Strelka2 [12] is supported as an alternative or complementary variant calling tool. Both Mutect2 and Strelka2 use an algorithm that models joint allele frequencies to call somatic variants [13] and both tools have been widely adopted by the scientific community. The support for both Mutect2 and Strelka2 allows for consensus variant calling by combining the results of both tools, a commonly used practice that yields more robust results. To distribute the workload in smaller subtasks, Halvade Somatic uses the same general principles as its predecessors that were designed for germline variant calling from DNA and RNA sequencing data [14, 15]: (i) the read alignment step can be parallelized by read, i.e., the process of aligning a particular read is independent of the alignment of another read; and (ii) preprocessing and variant calling steps are parallelized by genomic region, e.g., calling somatic variants in a particular genomic region is independent of variant calling in other regions. Compared with its counterparts for germline variant calling, Halvade Somatic is significantly more complex. First, the volume of data to process is larger due to the presence of two samples (tumor + normal) instead of only a single sample in case of germline variant calling. The data of both samples must be partitioned in a consistent manner across the parallel compute tasks while maintaining good load balance. Second, in order to have a good concordance between somatic variants called by the original (sequential) pipeline and the variants called by Halvade Somatic, we found that a careful design of the parallel base quality score recalibration (BQSR) step was essential: whereas BQSR for the germline variant calling pipeline could simply be applied to different genomic regions independently, the construction of *genome-wide* recalibration tables appears essential for somatic variant calling. Because of this, additional communication steps are required to aggregate locally computed, partial BQSR statistics into global statistics. Finally, whereas Halvade for germline variant calling was based on the MapReduce framework [16], Halvade Somatic is a re-implementation from scratch that leverages the Spark framework. Compared with MapReduce, Spark offers a richer framework with support for more complex communication and synchronization primitives as well as the ability to keep data in memory. As such, Spark is much better suited to deal with the different communication steps that arise from the parallelization of somatic variant calling pipelines.

Halvade Somatic is highly efficient: using a single 36-core compute node, runtime for the GATK/Mutect2 pipeline is reduced from ~84.5 h to ~19.5 h. This speedup of 4.3 × originates from a better utilization of the same hardware resources. Scaling to 16 nodes further reduces runtime to ~1 h 21 min, an additional speedup of ~14.4×, i.e., a total speedup of ~62.4× over the original pipeline. Users can select between the Mutect2 or Strelka2 variant callers or can choose to execute both tools, thus generating two separate variant callsets that can be combined and filtered to obtain high-confidence consensus variants [17]. Variant calling from both whole genome sequencing (WGS) as well as whole exome sequencing (WES) data is supported. To facilitate the execution of Halvade Somatic on a workstation without Spark installation, we provide a Docker image. Halvade Somatic can be executed on a wide variety of compute platforms, including the Amazon EC2.

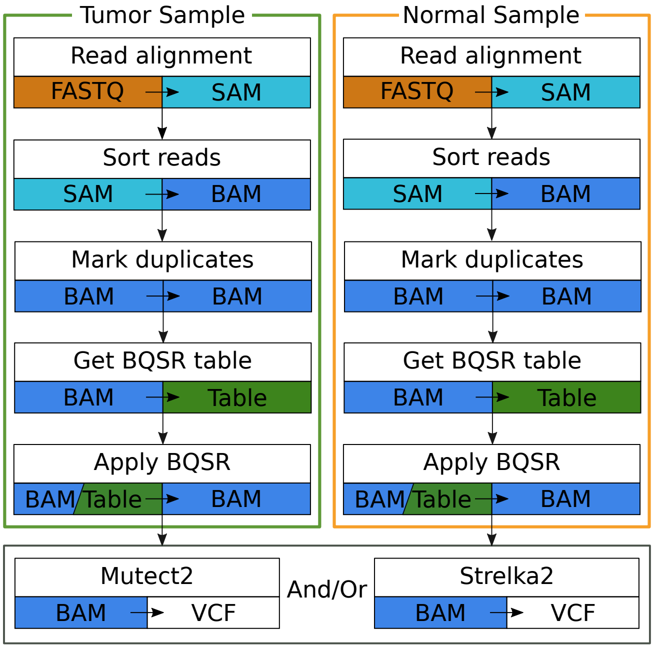

Figure 1. Somatic variant calling pipeline implemented in Halvade Somatic. Strelka2 can be run as an alternative or complementary tool to Mutect2.

Positioning with respect to state-of-the-art

As reviewed in [18], many bioinformatics workflows have been accelerated using Hadoop MapReduce or Spark. Tools such as BigBWA [19], SEAL [20] and Halvade [14] rely on the MapReduce programming model to accelerate sequence analysis pipelines. Whereas BigBWA and SEAL focus primarily on the read mapping phase, Halvade leverages MapReduce to accelerate an end-to-end germline GATK-based variant calling pipeline. The combination of parallel processing, the distributed-memory sorting functionality of Hadoop MapReduce and a scalable storage solution such as the Hadoop Distributed File System (HDFS) [21] yield efficient workflows that strongly reduce runtime. A more complex pipeline for germline variant calling from RNA-sequencing data was implemented in Halvade-RNA [15]. Halvade-RNA requires two successive MapReduce jobs to express the workflow. In between both MapReduce jobs, large volumes of intermediate data are stored on disk and loaded again when the second job commences.

The stringent map-sort-reduce paradigm as well as its disk-oriented processing are the main drawbacks of Hadoop MapReduce. The introduction of Spark solved these shortcomings and led to the introduction of a new generation of sequence analysis pipelines. SparkBWA [22] and StreamBWA [23] leverage Spark for the task of read mapping, whereas SparkGA [24, 25] implements a more comprehensive pipeline for germline variant calling according to the GATK best practices recommendations. A Spark-based adaption of an RNA-seq variant calling pipeline was provided by SparkRA [26].

These MapReduce and Spark-based workflows have in common that they execute, in parallel, multiple instances of *existing* tools (e.g., BWA [7] or GATK [9]) on subsets of the data. In other words, MapReduce and Spark are used to i) provide scalable I/O; ii) to manage parallel data streams and; iii) for task scheduling, synchronization and communication purposes. Most of the actual processing of sequencing data is done by existing tools. This modular approach makes it easy to integrate newer versions of these tools or to switch between alternative tools. For example, the elPrep [27] tool can be used as a drop-in replacement for certain modules of the GATK software suite. Alternatively, certain workflows such as ADAM/avocado [28] and certain GATK modules provide variant calling pipelines that are implemented in native Spark itself without relying on existing tools. However, such approach is rarely used as it requires extensive programming efforts.

In contrast to existing tools that focus on *germline* variant calling using either DNA-sequencing or RNA-sequencing data, we focus in this work on *somatic* variant calling from DNA-sequencing data. To the best of our knowledge, Halvade Somatic is the

first software framework to leverage a Big Data processing platform for this task.

Implementation

Apache Spark

Apache Spark is a data processing framework that was built to overcome some of the limitations of Hadoop MapReduce. Both frameworks share common principles such as the use of a distributed file system to provide scalable access to large volumes of data and support for parallel data processing in a fault tolerant manner. Compared to MapReduce, Apache Spark allows for a wider range of operations through its application programming interface (API) implemented in several programming languages. Additionally, Spark avoids disk I/O when possible: data is kept in memory for as long as possible and only written to disk when the data volume exceeds the memory capacity or when explicitly asked to persist data on disk. We briefly describe the most important terminology of Spark. For a more detailed account, we refer to [11].

Data is stored in Spark using *Resilient Distributed Datasets* (RDDs). RDDs can be thought of as containers for large volumes of data that are partitioned into smaller-sized chunks that are distributed over the local memories of the worker nodes. Operations on data are performed through *transformations* and *actions*. Transformations apply a particular operation to an RDD, yielding a new RDD which is again distributed over the worker nodes. In contrast, actions on RDDs apply operations for which the result is collected in the driver program.

Spark relies on *lazy evaluation* of RDDs. Subsequent transformations on RDDs form the Spark *lineage* which is evaluated only when an action is triggered. This is called a Spark *job*. The actual computations are performed by *executors*, i.e., processes on the worker nodes that are in charge of running individual tasks on subsets of the data. Lazy evaluation allows for performance optimizations as certain operations can be grouped together. Fault tolerance is provided by recomputing lost results: if an executor fails, the lineage is used to recalculate results starting from the last available data. When the data of an RDD is used multiple times and/or when the computation of an RDD is costly, it is beneficial to *persist* the RDD, which means that its data is explicitly stored in memory or on disk.

In order to execute existing tools (e.g. BWA or GATK) inside the Spark framework, we created a specialized `PipedRDD` implementation that supports common bioinformatics formats such as SAM (Sequence Alignment/Map) or BAM (Binary Sequence Alignment/Map) [29]. SAM records are represented as an iterator over strings while BAM data are represented as an array of bytes.

Halvade Somatic

Halvade Somatic leverages Apache Spark for the parallel, distributed-memory processing of the somatic variant calling pipeline shown in Fig. 1. A global overview of Halvade Somatic is depicted in Fig. 2. The workflow consists of three Spark jobs.

In the first job, the reads of the tumor DNA sample are aligned against the reference genome. Next, the reference genome is partitioned into N chromosomal regions in such a way that the regions contain roughly an equal number of aligned reads. In order to have an accurate, yet computationally efficient algorithm to determine the region boundaries, this procedure is performed on a randomly sampled subset of the aligned reads.

During the second job, the reads of the matched normal sample are aligned against the reference genome. The aligned read records of the tumor and normal samples are grouped according to the N regions that were established during the first job. Next, for each of the N regions independently, reads are sorted according to the position to which they align, read duplicates are marked and base quality score recalibration (BQSR) statistics are computed. These N partial BQSR statistics are aggregated into a single, genome-wide BQSR table.

Finally, in the third job, BQSR is applied to all reads. Somatic variants are called for each region independently. The N resulting partial Variant Call Format (VCF) files are merged into a single VCF output file.

Below, the different computational steps are described in more detail.

Input data preparation

Halvade Somatic supports input data as either unaligned reads (in FASTQ format) or as pre-aligned reads (in BAM format). In the former case, paired-end reads, per read group, are typically provided as two distinct, compressed (gzipped) FASTQ files. Halvade Somatic decompresses these files and splits them in smaller chunks (default size: 60 MB) that are distributed across worker nodes in such a way that paired-end reads are kept together. These chunks later serve as input for the alignment tasks that are executed in jobs 1 and 2 for the tumor and normal sample, respectively.

For performance reasons, the process of splitting data into chunk is multi-threaded. In case the data is provided as multiple read groups (and hence, multiple pairs of FASTQ files), this pre-processing step is performed by multiple Spark executors (i.e., multiple processes that are executed in parallel), one executor per read group. Often, the runtime of this pre-processing step is governed by data I/O.

Alternatively, the input data can be provided as pre-aligned BAM files. These BAM files are stored on the associated distributed file system (e.g. HDFS or Amazon S3) and parsed efficiently using Hadoop-BAM [30].

Read alignment, partitioning and sorting

Assuming unaligned input, multiple Spark executors run, in parallel, an instance of BWA [7] to align the reads of the tumor sample against the reference genome. Each BWA instance reads a FASTQ chunk from disk and streams the aligned SAM records to a `PipedRDD`. As the total number of FASTQ chunks is typically much higher than the number of executors, each executor has to process several chunks. Spark assigns chunks to executors such that the workload is evenly balanced while taking into account data locality. In case multiple CPU cores are assigned per executor, the multithreading functionality of BWA is used. The resulting RDD that holds the aligned SAM records is persisted as it is a dependency for multiple later steps. Since this RDD contains several hundreds of GB of data, it is persisted to disk by default.

Next, the reference genome is partitioned into N non-overlapping chromosomal regions. These regions will later be processed in parallel. The value of N is user-defined (default: 1800) and is typically much higher than the number of executors. The size of the chromosomal regions is non-uniform and is determined such that each region contains roughly the same number of aligned (tumor) reads. By accounting for possible variance in coverage among regions, we avoid regions with an excessive number of aligned reads and we obtain better load balancing compared to using uniformly-sized regions. For efficiency reasons, only a relatively small, randomly sampled subset of the tumor reads (default: 60N reads) is used to determine the size of the chromosomal regions. This action concludes the first Spark job.

In the second Spark job, the reads of the normal sample are aligned to the reference genome. The RDDs that contain the aligned tumor and normal reads are merged and partitioned according to the previously determined N chromosomal regions. This task requires the shuffling of large volumes of aligned read records and hence relies on inter-node communication. Read pairs that span the boundary of adjacent regions are duplicated in both regions.

After partitioning into regions, the reads are further sorted according to the chromosomal position to which they align. Data is spilled to disk if insufficient RAM is available, similar to how SAMtools [29] sorts SAM records. Sorted reads are written to BAM files on disk, one BAM file per chromosomal region.

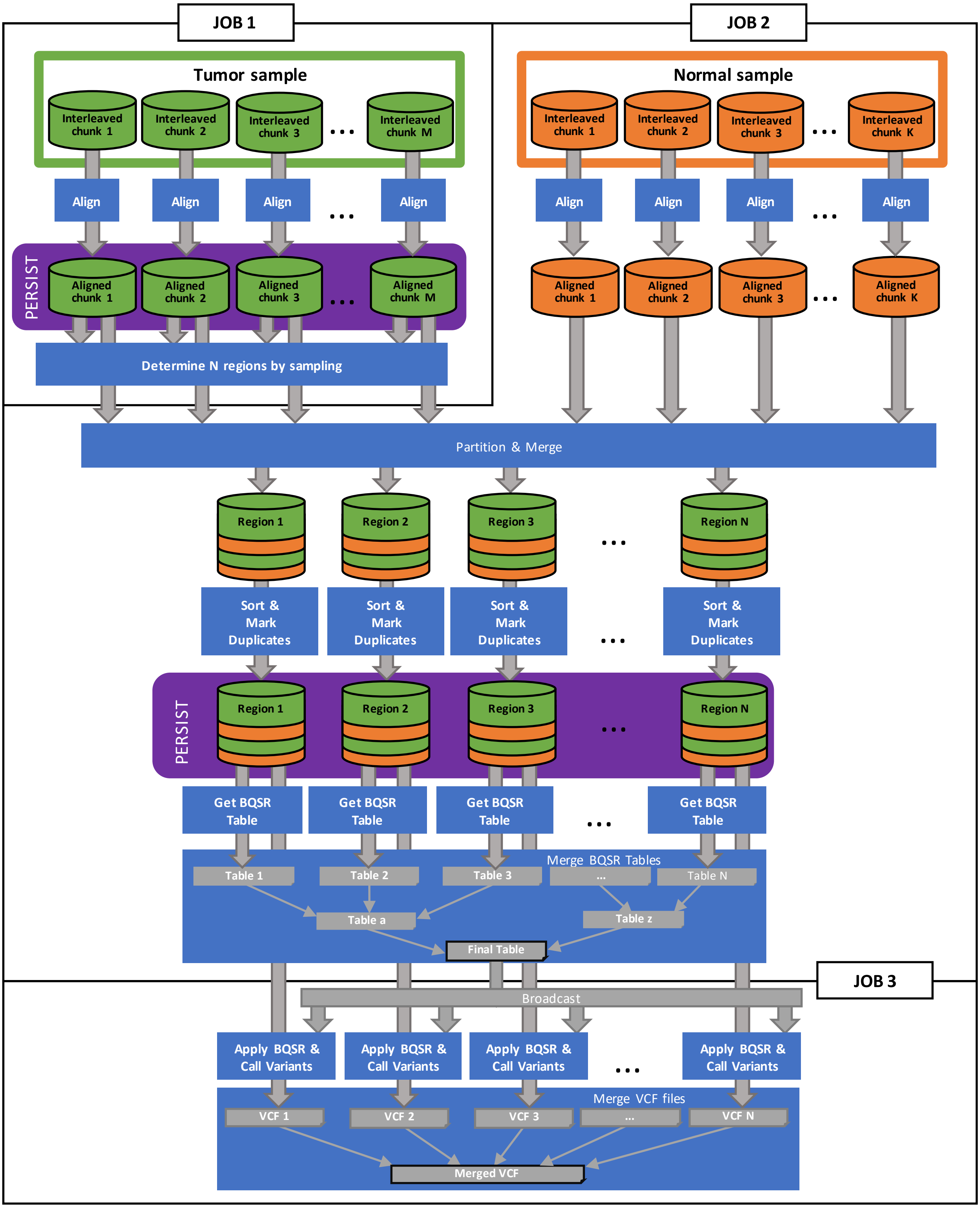

Figure 2. Overview of the Somatic variant calling framework in Spark. The workflow consists of three Spark jobs where the data at the end of jobs 1 and 2 are persisted. During the first job, the reads of the tumor sample are aligned to the reference genome and N chromosomal regions are determined such that each region contains roughly an equal number of aligned tumor reads. In the second job, the reads of the normal sample are aligned. The aligned reads (tumor and normal) are grouped per chromosomal region. Next, for each genomic region independently, reads are sorted according to the position to which they align and read duplicates are marked. This output is again persisted. Per genomic region, partial BQSR statistics are computed and merged into a genome-wide table. The last job uses this merged table to apply the BQSR to each read and call the somatic variants in all regions. The variants are merged into a single VCF output file.

Marking read duplicates, BQSR and variant calling

Polymerase chain reaction (PCR) and optical read duplicates cannot be considered as independent observations during variant calling and should therefore be marked accordingly. To this end, per chromosomal region independently, instances of the GATK 'Mark Duplicates' module are run. The resulting RDD is again persisted to disk. Our `PipedRDD` implementation supports running multiple instances of GATK per executor. This yields a significant performance increase when multiple CPU cores are assigned per executor and when the tool does not efficiently support multi-threading. Because GATK shows an average CPU usage of 100-200%, we assign one GATK instance per two CPU cores.

BQSR corrects for systematic errors when the sequencing machine estimates per-base quality scores. Per chromosomal region independently, a base quality score recalibration table is constructed using the 'BaseRecalibrator' module of GATK. A BQSR table summarizes empirically observed information on the quality score distribution and is required for the actual recalibration step. We avoid counting reads that span region boundaries (and that are present in both regions) twice.

Since the accuracy of the BQSR depends on the volume of the observed data and due to variability among the chromosomal regions we choose to aggregate these partial tables into a single *genome-wide* table using the `TreeReduce` action in Spark, concluding the second job. Even though this process requires inter-process communication and hence the synchronization of the different subtasks, we observed that merging the partial BQSR tables is essential to have a good correspondence between the variants called by the original (sequential) pipeline and those called by Halvade Somatic.

In the third Spark job, the merged BQSR table is distributed to all executors and the 'ApplyBQSR' module of GATK is executed. Finally, somatic variants are called using either Mutect2 or Strelka2, per chromosomal region independently, thus producing one Variant Call Format (VCF) file per chromosomal region that is stored using the Spark `saveAsTextFile` action. These partial VCF files are merged into a single VCF output file.

Optionally, if somatic variants from both Mutect2 and Strelka2 are desired, the BAM file that resulted from the BQSR step is persisted in order to avoid its recomputation. The second somatic variant caller is then run as a fourth Spark job (not shown in Fig. 2).

Spark Configuration

The correct configuration of the number of executors per worker node is essential for good performance. Note that the number of executors per node remains fixed across the different Spark jobs. In principle, a high number of executors is preferred to maximize parallelism in Spark. However, due to limited hardware resources, the number of executors is often restricted. When read mapping is required (FASTQ input), an executor requires ~16 GB RAM (8 GB for the BWA instance, 6 GB for the executor and 2 GB for executor overhead). This constraint often limits the number of executors. For example, for worker nodes with 64 GB of RAM, this translates into 4 executors per worker node. When the alignment step is not required (BAM input), the memory per executor can be reduced to about 1 GB per GATK instance, 6 GB for the executor and 2 GB overhead. The availability of more memory can improve performance in Spark as it reduces the chance of having to spill data to disk. The available CPU cores are evenly assigned to the different executors. We run multiple instances of a tool in parallel per executor if enough CPU cores are available. With this we can effectively increase CPU utilization and decrease overall runtime.

A second performance-critical parameter is the number of chromosomal regions N. More (and hence: smaller) regions lead to reduced memory requirements per executor but a higher tool starting overhead (e.g., GATK tries to check if it is running on a Google Cloud node, which can take several seconds). Additionally, a higher value of N increases the number of reads that need to be duplicated across adjacent regions. Nevertheless, using only few regions increases the volume of data per region, leading to increased memory requirements and difficulties to evenly balance the workload. From tests, we conclude that using 1500n to 1800n regions is optimal for a typical WGS sample and 250n to 320n for a typical WES sample. Here, n denotes the number of GATK

Table 1. Runtime of the original pipeline and Halvade Somatic for different combinations of samples (WGS or WES), input (FASTQ or BAM) and somatic variant calling tools (Mutect2, Strelka2 or both).

Sample	Input	Variant caller	Original pipeline (h)	Halvade Somatic (h)					
			1 node	1 node	2 nodes	4 nodes	8 nodes	12 nodes	16 nodes
WGS	FASTQ	Mutect2	84.57	19.45	9.35	4.81	2.47	1.74	1.36
WGS	FASTQ	Strelka2	55.66	18.74	9.19	4.45	2.31	1.57	1.21
WGS	FASTQ	Both	86.03	21.89	10.50	5.22	2.74	1.90	1.51
WGS	BAM	Mutect2	71.53	10.09	5.28	2.47	1.21	0.94	0.73
WGS	BAM	Strelka2	42.62	9.94	5.24	2.28	1.07	0.83	0.61
WGS	BAM	Both	72.99	12.77	6.91	2.99	1.53	1.13	0.96
WES	FASTQ	Mutect2	12.59	2.38	1.21				
WES	FASTQ	Strelka2	7.03	1.627	0.82				
WES	FASTQ	Both	12.66	2.65	1.36				
WES	BAM	Mutect2	10.72	1.70	0.85				
WES	BAM	Strelka2	5.16	0.86	0.42				
WES	BAM	Both	10.79	1.90	1.04				

instances per executor.

Results

Data and Availability

All whole genome sequencing (WGS) benchmarks were performed using 100 bp, paired-end Illumina HiSeq 2000 reads of a breast cancer sample (HCC1395) with a matched normal lymphoblastoid cell line (HCC1395 BL). Data are available through the Genome Modeling System (GMS) [31, 32] project and consists of about 1 billion reads (normal sample) and 1.88 billion reads (tumor sample), translating to sequencing depths of 34$\times$ and 62$\times$, respectively.

Whole exome sequencing (WES) benchmarks were performed using 100 bp, paired-end Illumina reads of the TCGA-A8-A08F sample. Data are available through the Cancer Genome Atlas Breast Invasive Carcinoma (TCGA-BRCA) data collection. The tumor sequencing data consists of 201 million reads, while the blood-derived normal sequencing data consists of about 156 million reads.

The Genome Reference Consortium Human build 38 (GRCh38) reference was used.

Performance Benchmarks

We first assess the computational performance of Halvade Somatic on a private computer cluster with 36 CPU cores (dual 2.30 GHz Intel(R) Xeon(R) Gold 6140 CPUs) and 187 GB of RAM per node. The worker nodes are connected to a General Parallel File System (GPFS) with a high-performance Enhanced Data Rate (EDR) Infiniband network. We used Spark 3.0.0, Hadoop Yarn 2.9.2, BWA 0.7.16a, Samtools 1.5, GATK 4.1.2.0 and Strelka 2.9.10. Halvade Somatic further relies on the HadoopBAM 7.10.0 and HtsJDK 2.11.0 libraries.

When input is provided as unaligned reads (FASTQ files), we use 9 executors per worker node, except for the worker node that also runs the Spark driver program, which has 8 executors. Each executor is thus allocated 4 CPU cores and ~20 GB of memory. Two instances of GATK are run per executor. When input is provided as aligned reads (BAM files), we use 18 executors per node (17 for the node that runs the driver), with 2 CPU cores and ~10 GB per executor. In that case, a single instance of GATK per executor is run.

Table 1 shows the runtimes of the original pipeline and Halvade Somatic for different combinations of input (FASTQ or BAM), different samples (WGS or WES) and somatic variant calling tools (Mutect2, Strelka2 or both). The original pipeline can be run only on a single node and multithreading was enabled for all tools that support it. Even on a single node, Halvade Somatic considerably

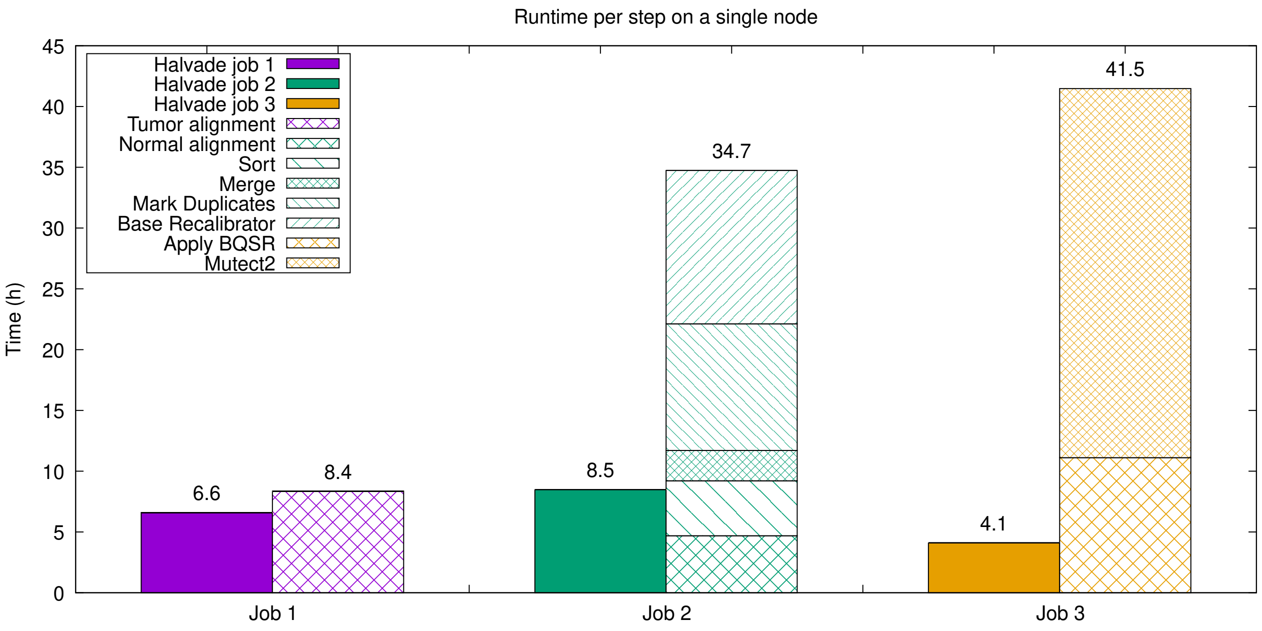

Figure 3. Comparison and breakdown of the runtime of Halvade Somatic and the original Mutect2 pipeline on a single node. Due to efficient multithreading support in BWA, the reduction in runtime for job 1 is limited. Jobs two and three show a significant reduction in runtime due to the limited support for multi-core architectures in GATK/Mutect2.

reduces the runtime: when Mutect2 is used as a somatic variant calling tool, runtime is reduced from 84.57 h to 19.45 h, a speedup of 4.34×. Fig. 3 shows a detailed breakdown of the runtime over the different steps. Clearly, the largest gains are obtained during Spark jobs 2 and 3, due to the under-utilization of hardware resources by GATK and Mutect2. Even though BWA has efficient support for multithreading, Halvade Somatic is able to slightly reduce the runtime of alignment steps as well.

Strelka2 is considerably faster than Mutect2 and has efficient support for multithreading. However, using a single node, Halvade Somatic is still 2.97× faster (55.66 h versus 18.74 h, see Table 1) for the entire pipeline. Running both Mutect2 and Strelka2 requires only little extra runtime compared to running only Mutect2. Hence, the use of both variant callers appears attractive to create a high-confidence set of somatic variants as also proposed in literature [17, 33].

When pre-aligned (BAM) input is provided, the alignment step can be omitted and runtime decreases accordingly. The relative gain from using Halvade Somatic is even more pronounced, as in this case, the pipeline predominantly consists of the GATK and Mutect2 steps. For example, when running both variant callers on a single node on the WGS dataset, the runtime is reduced from 72.99 h to 12.77 h (see Table 1), a speedup of 5.72×. Similarly, when using WES data, we observe, depending on input type and variant caller, speedups ranging from 4.32× to 6.31×.

For time-critical samples, Halvade Somatic can further reduce runtime by scaling to multiple worker nodes. Fig. 4 shows the parallel speedup obtained for the WGS sample and the different variant calling tools. The parallel speedup S_p is the ratio of runtime using a single node T_1 and the runtime using p nodes T_p. In the ideal case, S_p equals the number of nodes p. Due to communication and synchronization overhead, the observed speedups are slightly lower. Using 16 nodes and FASTQ input, we observe an additional parallel speedup that ranges between 14.4× (Mutect2 pipeline) and 15.4× (Strelka2 pipeline). This indicates that Halvade Somatic efficiently uses the extra hardware resources to reduce runtime. The combined effect of improved resource utilization of a node and the use of multiple nodes is significant: using the Mutect2 pipeline, the WGS sample and FASTQ input, runtime is reduced from 84.57 h (original pipeline, single node) to 1.36 h (Halvade Somatic, 16 nodes), an overall speedup of 62.4×. Similarly, using the Strelka2 variant caller, runtime is reduced from 55.66 h (original pipeline, single node) to 1.21 h (Halvade Somatic, 16 nodes).

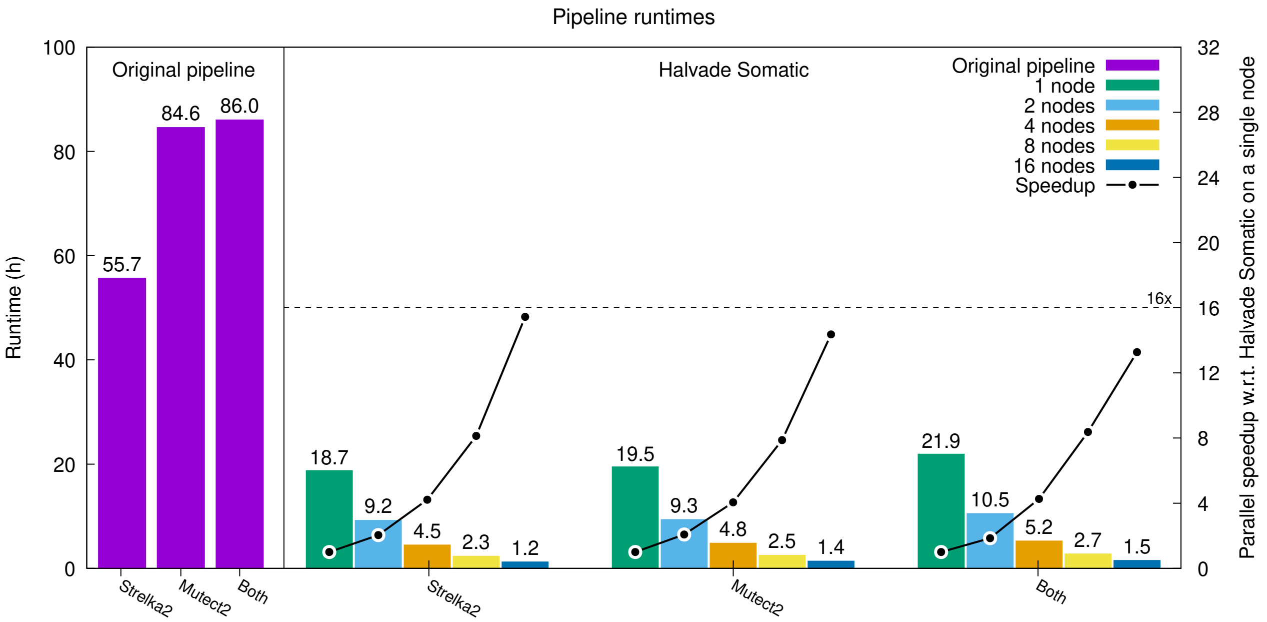

Figure 4. Runtime and parallel speedup for the WGS sample using FASTQ input.

Table 2. Runtime of Halvade somatic for the Mutect2 pipeline on Amazon EMR. The cost is calculated using standard pricing of region eu-west-1 (Ireland) at the time of writing.

Sample	Input	Number of nodes	Halvade Somatic runtime (h)	Cost (USD)
WGS	FASTQ	8	3.25	83.81
WGS	BAM	8	1.43	41.90
WES	FASTQ	1	2.75	8.80
WES	FASTQ	2	1.42	11.02
WES	BAM	1	1.88	5.87
WES	BAM	2	1.08	11.02

Cloud and Docker Support

Docker image

We provide a Docker image to facilitate the deployment of Halvade Somatic on a node without native Spark installation. The image contains all necessary software packages and libraries.

The use of a Docker image imposes virtually no computational overhead: using a node with 32 CPU cores (dual 2.30 GHz Intel(R) Xeon(R) CPU E5-2698 v3) and 256 GB of memory, we measured a runtime for the WGS sample of 20.59 h and 9.55 h for FASTQ and BAM input, respectively. For the WES sample we measured a runtime of 2.10 h and 1.25 h for FASTQ and BAM input, respectively.

Amazon EMR

Halvade Somatic can also be deployed on public cloud compute platforms such as Amazon EMR. Input data, reference files, binaries and libraries should be uploaded to Amazon S3 storage. We provide a bootstrap script to copy certain files from Amazon S3 storage to the individual worker nodes, a task that requires about 10 minutes. We benchmarked Halvade Somatic using an r5d.xlarge node (2 CPU cores, 32 GB of RAM and a single 150 GB NVMe SSD) to run the driver program and r5d.8xlarge nodes (16 CPU cores, 256 GB of RAM and 2 $\times$ 600 GB NVMe SSDs) as worker nodes. The runtime of Halvade Somatic for the Mutect2 pipeline is shown in Table 2 for the different samples, input type and a different number of nodes, along with the total financial cost using standard Amazon pricing.

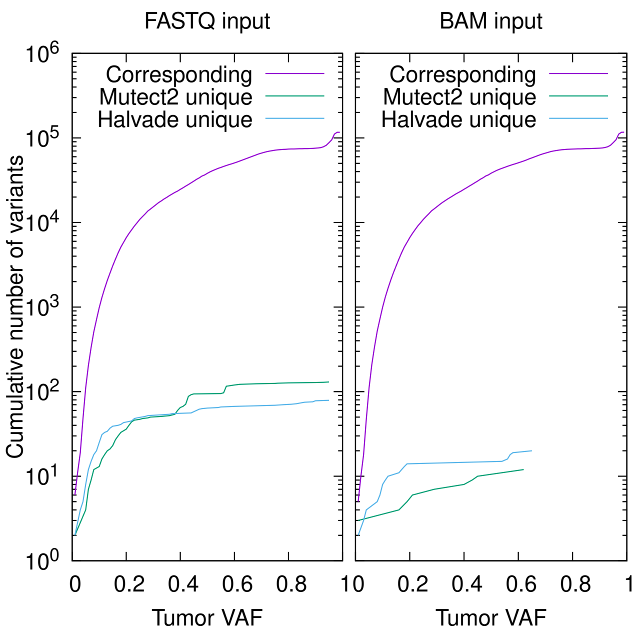

Figure 5. Cumulative number of corresponding and discordant somatic variants between the original GATK/Mutect2 pipeline and Halvade Somatic as a function of the tumor variant allele frequency (VAF).

Assessment of Variant Accuracy

The resulting VCF file can differ slightly between a parallelized Halvade Somatic run and the corresponding sequential pipeline. We emphasize that the set of somatic variants called by the original pipeline most likely does not fully correspond to the biological ground truth and suffers from false positive and false negative variants. It is well-known that somatic variant calling is a notoriously difficult problem and different somatic variant calling tools often show limited overlap in their output (see e.g. [34]). In this section, we pinpoint the origins of the small differences that arise purely due to the parallelization of the pipeline itself.

Using the original GATK/Mutect2 pipeline and WGS data, we find 116 791 somatic variants after filtering with the GATK 'FilterMutectCalls' module. Starting from FASTQ input, Halvade Somatic identifies 116 661 overlapping (99,89%), 130 missed (0,11%) and 79 additional (0.07%) somatic variants (see Fig. 5). Most of these differences are due to differential read alignment: because of parallelization, the order in which (paired-end) reads are presented to BWA causes output differences. This is due to the random placement of repetitive reads and the fact that the fragment size of paired-end reads may be estimated slightly different for different FASTQ chunks. To confirm this, we ran the original GATK/Mutect2 pipeline on a shuffled FASTQ file and observed the same degree of variability in resulting somatic variants (data not shown).

When using Halvade Somatic with pre-aligned BAM input, we eliminate this source of variation and identify 116 779 overlapping (99,99%), 12 missed (0,01%) and 20 additional (0.017%) somatic variants. These small differences in output are caused by subtle variability during the mark duplicates step that may occur for reads that span region boundaries. Additionally, Mutect2 uses random downsampling at positions with extremely high coverage.

We conclude that the variants called by Halvade Somatic match those of the original pipeline to a very high degree and that very small differences in output are mostly due to random effects.

Discussion and Conclusion

The accurate identification of somatic variants from NGS data is time consuming, especially when WGS data is used. Individual tools for read mapping, data preparation and variant calling have matured, but often lack support for multi-node and sometimes

even multi-core computer systems. This, in turn, translates to high execution times –often days– to process raw sequencing data. For germline variant calling several software tools have been proposed in literature that leverage big data platforms such as MapReduce or Spark to strongly reduce runtime. For the problem of somatic variant calling, however, such tools are lacking.

Halvade Somatic implements the somatic variant calling pipeline according to the GATK best practices recommendations. It supports both the Mutect2 and Strelka2 variant callers and takes advantage of the Apache Spark framework to call somatic variants with high computational performance, scalability and reliability. Most of the workload can be parallelized: reads can be mapped in parallel while data preprocessing steps and variant calling can be parallelized by genomic region. Spark is used to create and manage parallel data streams and run multiple instances of tools in parallel on subsets of the data. To partition and sort aligned SAM records, and to build a genome-wide BQSR table, we rely on Spark communication primitives to exchange the relevant data among worker nodes.

Halvade Somatic drastically reduces runtimes even on a single node: depending on the exact setup (WES or WGS, FASTQ or BAM input, choice of variant caller), we measured a speedup ranging from 2.97$\times$ to 6.31$\times$. Halvade also scales very well across multiple nodes if a larger cluster is available. We observe parallel speedups of 13.27$\times$ and higher when scaling to 16 nodes.

Source code is available under the GPL v3.0 license at https://bitbucket.org/dries_decap/halvadeforspark/. Extensive documentation is available online (https://halvadeforspark.readthedocs.io). A Docker image is provided to run Halvade on a single node. Cloud support is available through Amazon EMR.

Availability of source code and requirements

- Project name: Halvade Somatic
- Project home page: https://bitbucket.org/dries_decap/halvadeforspark/src/master/
- Operating system(s): Linux
- Programming language: Scala
- Other requirements: Apache Spark 3.0 or higher, GATK 4.1.2.0 or higher, Samtools 1.5 or higher and BWA 0.7.16 or higher
- License: GPL v3.0

Declarations

List of abbreviations

API: Application Programming Interface; BAM: Binary Sequence Alignment/Map; BQSR: Base Quality Score Recalibration; BWA: Burrows-Wheeler Aligner; CCLE: Cancer Cell Line Encyclopedia; CPU: Central Processing Unit; EDR: Enhanced Data Rate; GATK: Genome Analysis ToolKit; GPFS: General Parallel File System; HCC: Human Cancer Cell Line; HDFS: Hadoop Distributed File System; GRCh38: Genome Reference Consortium Human build 38; ICGC: International Cancer Genome Consortium; NGS: Next Generation Sequencing; PCR: Polymerase Chain Reaction; RAM: Random-Access Memory; RDD: Resilient Distributed Datasets; SAM: Sequence Alignment/Map Format; TCGA: The Cancer Genome Atlas Program; TCGA-BRCA: Cancer Genome Atlas Breast Invasive Carcinoma; VAF: Variant Allele Frequency; VCF: Variant Call Format; WGS: Whole Genome Sequencing; WES: Whole Exome Sequencing;

Consent for publication

Not applicable.

Competing Interests

The author(s) declare that they have no competing interests.

Funding

This research is conducted within the project entitled 'ATHENA – Augmenting Therapeutic Effectiveness through Novel Analytics', project no. HBC.2019.2528, funded by VLAIO (Flanders Innovation & Entrepreneurship).

Author's Contributions

D.D. designed and developed Halvade Somatic. P.C., C.H. and R.W. assisted with the performance analysis. L.S.B., M.L., and K.M. aided with the accuracy assessment. J.F. supervised the work. D.D. and J.F. wrote the manuscript. All authors read and approved the manuscript.

Availability of supporting data and materials

The human genome reference GRCh38 and all required reference files used in this article are publicly available through the Resource bundle of GATK at https://console.cloud.google.com/storage/browser/genomics-public-data/resources/broad/hg38/v0/. We used the *Homo_sapiens_assembly38.fasta* reference file and *Homo_sapiens_assembly38.known_indels.vcf.gz* which contains the known variants. The HCC1395 WGS sample [31] used in all benchmarks in this paper is publicly available as well at http://genomedata.org/pmbio-workshop/fastqs/all/. The WES sample are available through the TCGA-BRCA data collection at https://portal.gdc.cancer.gov/cases/0a017f15-1c6b-45e7-8d55-e0a71df1b2e8. Detailed documentation to run and use Halvade is available at https://halvadeforspark.readthedocs.io/en/latest.

Acknowledgements

The computational resources (Stevin Supercomputer Infrastructure) and services used in this work were provided by the VSC (Flemish Supercomputer Center), funded by Ghent University, FWO and the Flemish Government – department EWI.

References

1. The Cancer Genome Atlas Research Network, Weinstein JN, Collisson EA, Mills GB, Shaw KR, Ozenberger BA, et al. The Cancer Genome Atlas Pan-Cancer analysis project. Nature Genetics 2013 Oct;45(10):1113–1120. https://doi.org/10.1038/ng.2764.
2. Barretina J, Caponigro G, Stransky N, Venkatesan K, Margolin AA, Kim S, et al. The Cancer Cell Line Encyclopedia enables predictive modelling of anticancer drug sensitivity. Nature 2012;483:603–607. https://doi.org/10.1038/nature11003.
3. Zhang J, Bajari R, Andric D, Gerthoffert F, Lepsa A, Nahal-Bose H, et al. The International Cancer Genome Consortium Data Portal. Nature Biotechnology 2019;37:367–369. https://doi.org/10.1038/s41587-019-0055-9.
4. Dagogo-Jack I, Shaw AT. Tumour heterogeneity and resistance to cancer therapies. Nature Reviews Clinical Oncology 2018;15(2):81–94. https://doi.org/10.1038/nrclinonc.2017.166.
5. Illumina. Evaluating Somatic Variant Calling in Tumor/Normal Studies. Illumina, Inc; 2014.
6. Van der Auwera GA, O'Connor BD. Genomics in the Cloud: Using Docker, GATK, and WDL in Terra. CA 95472 Sebastopol, Canada: O'Reilly Media, Inc; 2020.

7. Li H, Durbin R. Fast and accurate short read alignment with Burrows-Wheeler transform. Bioinformatics 2009 May;25(14):1754–1760. https://doi.org/10.1093/bioinformatics/btp324.
8. Broad Institute, Picard Tools; 2021. Accessed: 2021-07-26. http://broadinstitute.github.io/picard/.
9. McKenna A, Hanna M, Banks E, Sivachenko A, Cibulskis K, Kernytsky A, et al. The Genome Analysis Toolkit: a MapReduce framework for analyzing next-generation DNA sequencing data. Genome research 2010;20(9):1297–1303. https://doi.org/10.1101/gr.107524.110.
10. Cibulskis K, Lawrence MS, Carter SL, Sivachenko A, Jaffe D, Sougnez C, et al. Sensitive detection of somatic point mutations in impure and heterogeneous cancer samples. Nature biotechnology 2013;31(3):213. https://doi.org/10.1038/nbt.2514.
11. Zaharia M, Chowdhury M, Franklin MJ, Shenker S, Stoica I. Spark: Cluster Computing with Working Sets. In: Proceedings of the 2nd USENIX Conference on Hot Topics in Cloud Computing HotCloud'10, USA: USENIX Association; 2010. p. 10.
12. Scheffler K, Halpern AL, Bekritsky MA, Noh E, Källberg M, Chen X, et al. Strelka2: fast and accurate calling of germline and somatic variants. Nature Methods 2018;15:591–594. https://doi.org/10.1038/s41592-018-0051-x.
13. Xu C. A review of somatic single nucleotide variant calling algorithms for next-generation sequencing data. Computational and Structural Biotechnology Journal 2018;16:15–24. https://www.sciencedirect.com/science/article/pii/S2001037017300946.
14. Decap D, Reumers J, Herzeel C, Costanza P, Fostier J. Halvade: scalable sequence analysis with MapReduce. Bioinformatics 2015;31(15):2482–2488. http://dx.doi.org/10.1093/bioinformatics/btv179.
15. Decap D, Reumers J, Herzeel C, Costanza P, Fostier J. Halvade-RNA: Parallel variant calling from transcriptomic data using MapReduce. PLOS ONE 2017 03;12(3):1–11. https://doi.org/10.1371/journal.pone.0174575.
16. Dean J, Ghemawat S. MapReduce: Simplified Data Processing on Large Clusters. Commun ACM 2008 Jan;51(1):107–113. https://doi.org/10.1145/1327452.1327492.
17. Wang M, Luo W, Jones K, Bian X, Williams R, Higson H, et al. SomaticCombiner: improving the performance of somatic variant calling based on evaluation tests and a consensus approach. Scientific Reports 2020 07;10:12898. https://doi.org/10.1038/s41598-020-69772-8.
18. Guo R, Zhao Y, Zou Q, Fang X, Peng S. Bioinformatics applications on Apache Spark. GigaScience 2018 08;7(8). https://doi.org/10.1093/gigascience/giy098, giy098.
19. Abuín JM, Pichel JC, Pena TF, Amigo J. BigBWA: approaching the Burrows–Wheeler aligner to Big Data technologies. Bioinformatics 2015 08;31(24):4003–4005. https://doi.org/10.1093/bioinformatics/btv506.
20. Pireddu L, Leo S, Zanetti G. SEAL: a distributed short read mapping and duplicate removal tool. Bioinformatics 2011 06;27(15):2159–2160. https://doi.org/10.1093/bioinformatics/btr325.
21. Shvachko K, Kuang H, Radia S, Chansler R. The Hadoop Distributed File System. In: 2010 IEEE 26th Symposium on Mass Storage Systems and Technologies (MSST); 2010. p. 1–10. https://doi.org/10.1109/MSST.2010.5496972.
22. Abuín JM, Pichel JC, Pena TF, Amigo J. SparkBWA: Speeding Up the Alignment of High-Throughput DNA Sequencing Data. PLOS ONE 2016 05;11(5):1–21. https://doi.org/10.1371/journal.pone.0155461.
23. Mushtaq H, Ahmed N, Al-Ars Z. Streaming Distributed DNA Sequence Alignment Using Apache Spark. In: 2017 IEEE 17th International Conference on Bioinformatics and Bioengineering (BIBE); 2017. p. 188–193. https://doi.org/10.1109/BIBE.2017.00-57.
24. Mushtaq H, Liu F, Costa C, Liu G, Hofstee P, Al-Ars Z. SparkGA: A Spark Framework for Cost Effective, Fast and Accurate DNA Analysis at Scale. In: Proceedings of the 8th ACM International Conference on Bioinformatics, Computational Biology,and Health Informatics ACM-BCB '17, New York, NY, USA: Association for Computing Machinery; 2017. p. 148–157. https://doi.org/10.1145/3107411.3107438.
25. Mushtaq H, Ahmed N, Al-Ars Z. SparkGA2: Production-quality memory-efficient Apache Spark based genome analysis frame-

work. PLOS ONE 2019 12;14(12):1–14. https://doi.org/10.1371/journal.pone.0224784.

26. Al-Ars Z, Wang S, Mushtaq H. SparkRA: Enabling Big Data Scalability for the GATK RNA-seq Pipeline with Apache Spark. Genes 2020;11(1). https://www.mdpi.com/2073-4425/11/1/53.

27. Herzeel C, Costanza P, Decap D, Fostier J, Wuyts R, Verachtert W. Multithreaded variant calling in elPrep 5. PLOS ONE 2021 02;16(2):1–13. https://doi.org/10.1371/journal.pone.0244471.

28. Massie M, Nothaft F, Hartl C, Kozanitis C, Schumacher A, Joseph AD, et al. ADAM: Genomics Formats and Processing Patterns for Cloud Scale Computing. EECS Department, University of California, Berkeley; 2013.

29. Li H, Handsaker B, Wysoker A, Fennell T, Ruan J, Homer N, et al. The Sequence Alignment/Map format and SAMtools. Bioinformatics 2009 Jun;25(16):2078–2079. https://doi.org/10.1093/bioinformatics/btp352.

30. Niemenmaa M, Kallio A, Schumacher A, Klemelä P, Korpelainen E, Heljanko K. Hadoop-BAM: directly manipulating next generation sequencing data in the cloud. Bioinformatics 2012;28(6):876–877. https://doi.org/10.1093/bioinformatics/bts054.

31. Griffith M, Griffith OL, Smith SM, Ramu A, Callaway MB, Brummett AM, et al. Genome modeling system: a knowledge management platform for genomics. PLoS computational biology 2015;11(7). https://doi.org/10.1371/journal.pcbi.1004274.

32. The Genome Modeling System;. Accessed: 2021-07-26. https://github.com/genome/gms/wiki.

33. de Schaetzen van Brienen L, Larmuseau M, Van der Eecken K, De Ryck F, Robbe P, Schuh A, et al. Comparative analysis of somatic variant calling on matched FF and FFPE WGS samples. BMC Medical Genomics 2020 July;13(1):94. https://doi.org/10.1186/s12920-020-00746-5.

34. Cai L, Yuan W, Zhang Z, He L, Chou KC. In-depth comparison of somatic point mutation callers based on different tumor next-generation sequencing depth data. Scientific reports 2016;6:36540. https://doi.org/10.1038/srep36540.

Cover Letter Click here to access/download;Personal Cover;Cover Letter.pdf

FACULTY OF ENGINEERING AND ARCHITECTURE

DEPARTMENT OF INFORMATION TECHNOLOGY
RESEARCH GROUP IDLAB

Jan Fostier
Associate Professor

E jan.fostier@ugent.be
T +32 9 331 48 75
F +32 9 331 49 00

iGent Tower, floor 9
Technologiepark-Zwijnaarde 126
B-9052 Gent
Belgium

GigaScience
Oxford University Press
Walton Street
Oxford

DATE	PAGE	OUR REFERENCE
1 September 2021	1/1	Halvade Somatic

Dear Editor,

Please find enclosed our manuscript entitled "Halvade Somatic: Somatic Variant Calling with Apache Spark", submitted as a Technical Note to GigaScience.

In this work, we propose Halvade Somatic, the first pipeline to leverage Big Data platforms for somatic variant calling. It is based on the GATK / Mutect2 pipeline. Using Apache Spark, we provide a scalable and reliable pipeline that strongly reduces runtime. For example, runtime is reduced from 84.5h (single 36-core node with original pipeline) to 1h 21min (a cluster of 16 nodes with Halvade Somatic), a speedup of 62.4x.

Halvade Somatic also supports the use of Strelka2 as an alternative of complementary variant calling tool, and thus allows for consensus variant calling. We support both whole genome and whole exome sequencing data using either FASTQ or BAM input. We demonstrate its ability to run on public cloud infrastructure such as Amazon EMR. A Docker container is provided to facilitate the deployment of Halvade Somatic on a single node without local Spark installation.

Our software is available as open source under GPL 3.0 license. Extensive online documentation is available online.

On behalf of all authors, I thank you for considering our manuscript for publication.

Kind regards,

Jan Fostier

GHENT UNIVERSITY

imec
