# Supplementary material for: Halvade somatic: Somatic variant calling with Apache Spark
Source: Gigascience. 2022 Jan 12;11:giab094. doi: 10.1093/gigascience/giab094 (PMC8756192; doi:10.1093/gigascience/giab094)
Supplement: giab094_Response_to_Reviewer_Comments_Original_Submission [file giab094_response_to_reviewer_comments_original_submission.pdf]

Author's Response To Reviewer Comments

Close

Dear Editor,

Thank you for handling our manuscript.

We have added the sentence and reference [35] to section "Availability of supporting data and materials" as per your instructions. Kindly note that the doi link is not (yet) working.

Kind regards,

Jan Fostier

Close
