# Supplementary material for: Halvade somatic: Somatic variant calling with Apache Spark
Source: Gigascience. 2022 Jan 12;11:giab094. doi: 10.1093/gigascience/giab094 (PMC8756192; doi:10.1093/gigascience/giab094)
Supplement: giab094_Reviewer_1_Report_Original_Submission — Medhat Mahmoud -- 9/27/2021 Reviewed [file giab094_reviewer_1_report_original_submission.pdf]

Reviewer Report

Title: Halvade Somatic: Somatic Variant Calling with Apache Spark

Version: Original Submission **Date:** 9/27/2021

Reviewer name: Medhat Mahmoud

Reviewer Comments to Author:

Identification of somatic variants is of great importance. The tools designed to identify these variants are time-consuming. Decap et al., in their paper "Halvade Somatic: Somatic Variant Calling with Apache Spark" introduces a new method to the parallel running of somatic variant caller likewise aligner (BWA, GATK, Mutect2, and Strelka2) using Apache spark.
I enjoyed reading the paper. The introduction sections explain the motivation, the how, and the methods used especially Apache spark, and compare to the previous start of the art tools.
With that said;
I do not know If I'm missing this part, or it did not exist; the authors did not discuss how the user can control the parameters of each step? (aligning and calling variant)
Splitting reads for aligning is not recommended, and from figure 3 run time was not reduced much compared to jobs 2 and 3, so why the authors did not just align all reads without splitting?
What shall happen to the reads aligned near the boundaries of partitioned regions?
Figure 5, could be enhanced the authors mentioned corresponding and discordant somatic variants, which is not clear from the figure. Maybe you could associate a bar chart showing a benchmark between results from Halvade and the other tools.
Thank you,

Level of Interest

Please indicate how interesting you found the manuscript: Choose an item.

Quality of Written English

Please indicate the quality of language in the manuscript: Choose an item.

Declaration of Competing Interests

Please complete a declaration of competing interests, considering the following questions:

- Have you in the past five years received reimbursements, fees, funding, or salary from an organisation that may in any way gain or lose financially from the publication of this manuscript, either now or in the future?

- Do you hold any stocks or shares in an organisation that may in any way gain or lose financially from the publication of this manuscript, either now or in the future?
- Do you hold or are you currently applying for any patents relating to the content of the manuscript?
- Have you received reimbursements, fees, funding, or salary from an organization that holds or has applied for patents relating to the content of the manuscript?
- Do you have any other financial competing interests?
- Do you have any non-financial competing interests in relation to this paper?

If you can answer no to all of the above, write 'I declare that I have no competing interests' below. If your reply is yes to any, please give details below.

I declare that I have no competing interests

I agree to the open peer review policy of the journal. I understand that my name will be included on my report to the authors and, if the manuscript is accepted for publication, my named report including any attachments I upload will be posted on the website along with the authors' responses. I agree for my report to be made available under an Open Access Creative Commons CC-BY license (http://creativecommons.org/licenses/by/4.0/). I understand that any comments which I do not wish to be included in my named report can be included as confidential comments to the editors, which will not be published.

Choose an item.

To further support our reviewers, we have joined with Publons, where you can gain additional credit to further highlight your hard work (see: https://publons.com/journal/530/gigascience). On publication of this paper, your review will be automatically added to Publons, you can then choose whether or not to claim your Publons credit. I understand this statement.

Yes Choose an item.
