# Supplementary material for: Halvade somatic: Somatic variant calling with Apache Spark
Source: Gigascience. 2022 Jan 12;11:giab094. doi: 10.1093/gigascience/giab094 (PMC8756192; doi:10.1093/gigascience/giab094)
Supplement: giab094_Reviewer_2_Report_Original_Submission — Jiarui Ding, Ph.D. -- 10/2/2021 Reviewed [file giab094_reviewer_2_report_original_submission.pdf]

Reviewer Report

Title: Halvade Somatic: Somatic Variant Calling with Apache Spark

Version: Original Submission **Date:** 10/2/2021

Reviewer name: Jiarui Ding, Ph.D.

Reviewer Comments to Author:

In this paper, Decap and co-authors developed Hlvade Somatic, an efficient somatic mutation call pipeline for pair-normal-tumor second-generation sequencing data. Somatic mutation call is an essential task for cancer genomics studies, thus making this time-consuming step more efficient is of great help to the community. The manuscript is well written and easy to follow. I only have a few comments for the authors to address:
1, I'm wondering the limits of the pipeline: given the almost unlimited number of CPUs such as Google cloud, how fast can we get the mutation calls for a pair of human genomes? What can we do to push the limits?
2, Given limited resources, for example, a cluster with 64 cores, how to optimally set the many parameters? I suggest the authors provide more friendly tutorials for such use cases to guide interested readers to use the pipeline.
3, What is the percentage of extra energy (e.g., electricity) by using this highly parallel processing compared to just one core? There are still many cases where time is not critical for mutation calls.
4, Is it possible for the authors to set up everything in the Google cloud, and users can upload data to run the pipeline and then download results? Of course, the users will pay the costs.

Level of Interest

Please indicate how interesting you found the manuscript: Choose an item.

Quality of Written English

Please indicate the quality of language in the manuscript: Choose an item.

Declaration of Competing Interests

Please complete a declaration of competing interests, considering the following questions:

- Have you in the past five years received reimbursements, fees, funding, or salary from an organisation that may in any way gain or lose financially from the publication of this manuscript, either now or in the future?
- Do you hold any stocks or shares in an organisation that may in any way gain or lose financially from the publication of this manuscript, either now or in the future?

- Do you hold or are you currently applying for any patents relating to the content of the manuscript?
- Have you received reimbursements, fees, funding, or salary from an organization that holds or has applied for patents relating to the content of the manuscript?
- Do you have any other financial competing interests?
- Do you have any non-financial competing interests in relation to this paper?

If you can answer no to all of the above, write 'I declare that I have no competing interests' below. If your reply is yes to any, please give details below.

I declare that I have no competing interests

I agree to the open peer review policy of the journal. I understand that my name will be included on my report to the authors and, if the manuscript is accepted for publication, my named report including any attachments I upload will be posted on the website along with the authors' responses. I agree for my report to be made available under an Open Access Creative Commons CC-BY license (http://creativecommons.org/licenses/by/4.0/). I understand that any comments which I do not wish to be included in my named report can be included as confidential comments to the editors, which will not be published.

Choose an item.

To further support our reviewers, we have joined with Publons, where you can gain additional credit to further highlight your hard work (see: https://publons.com/journal/530/gigascience). On publication of this paper, your review will be automatically added to Publons, you can then choose whether or not to claim your Publons credit. I understand this statement.

Yes Choose an item.
