# Supplementary material for: Halvade somatic: Somatic variant calling with Apache Spark
Source: Gigascience. 2022 Jan 12;11:giab094. doi: 10.1093/gigascience/giab094 (PMC8756192; doi:10.1093/gigascience/giab094)
Supplement: giab094_Reviewer_3_Report_Original_Submission — Zaid Al-Ars -- 10/16/2021 Reviewed [file giab094_reviewer_3_report_original_submission.pdf]

Reviewer Report

Title: Halvade Somatic: Somatic Variant Calling with Apache Spark

Version: Original Submission **Date:** 10/16/2021

Reviewer name: Zaid Al-Ars

Reviewer Comments to Author:

This work describes a scalable and distributed somatic variant calling pipeline using the Apache Spark framework. The pipeline is based on the tools used in the GATK best practices recommendations.
The article is well written and well illustrated. However there are minor and major concerns as discussed below.
Minor concerns and issues

We observed following issues/comments. Please take them into consideration to add value to this article:
1. As Mutect2 and Strelka2 don't support region based variant calling, Fig. 2, doesn't show how a chromosomal region-specific BAM file is being processed and how the reference genome in this step is being distinguished for that particular region/chunk being processed. Can you please clarify this issue?
2. Section "Read alignment, partitioning and sorting", Line 172: Is this statement correct? "Next, the reference genome is partitioned into N non-overlapping chromosomal regions. These regions will later be processed in parallel." It Is better to structure the sections in the paper in the same way as shown in Fig. 2: "Align, partition and merge", "Sort and mark duplicates", and "BQSR and variant calling". This makes the discussion easier to follow.
3. Section "Marking read duplicates, BQSR and variant calling", Line 188: "To this end, per chromosomal region independently, instances of the GATK 'Mark Duplicates' module are run." I think you referent to Picard Mark Duplicates and not GATK?
4. Section "Assessment of Variant Accuracy", Line 296: "Most of these differences are due to differential read alignment: because of parallelization, the order in which (paired-end) reads are presented to BWA causes output differences. This is due to the random placement of repetitive reads and the fact that the fragment size of paired-end reads may be estimated slightly different for different FASTQ chunks." However, in Section "Input data preparation" the paper claims to compensate for this effect "Halvade Somatic decompresses these files and splits them in smaller chunks (default size: 60 MB) that are distributed across worker nodes in such a way that paired-end reads are kept together." Please clarify what then is the effect of keeping pair-ends together? Does this increase the accurace? And if so, where and why do difference still occur?
5. Section "Assessment of Variant Accuracy", Line 302: "These small differences in output are caused by subtle variability during the mark duplicates step that may occur for reads that span region boundaries." This is usually what we assume as a cause for reduction in accuracy. However, it is mentioned here as a fact. Do you have any strong evidence to prove this assumption? Did you perform any experiments to show this?

6. Whole exome sequencing (WES) benchmarks datasets source/reference is not provided. Also please mentioned the coverage depth.
7. How many BWA instance on each node are being initiated?
8. What is the feasibility to integrate any other (latest/more accurate) somatic variant caller like Octopus to this framework? Or is this framework limited to the discussed variant callers?
9. In Fig. 2, at step 2 output and step 3 input only BQSR tables are mentioned. No mention of BAM files and how the reference genome is being used in variant calling steps. What is the data format used? Is the output backed up to disk in any specific format or is it only being used actively in memory.

Major concerns

At the same time, we have following major concerns on the novelty of this work.
1. Low novelty level for a high quality journal such as GigaScience. There is limited novelty in this work, with the main contribution is using existing the Spark-based Halvade framework to process tumor datasets with Mutect2 and Strelka2 variant callers instead of GATK Haplotypecaller for germinal datasets. There needs to be some more conceptual contribution wither to the algorithmic approach, or to the utilized frameworks, or to the used hardware infrastructure, or to the used libraries or data representations. At the moment all of these are missing.
2. The authors use single-threaded Picard and GATK applications to run their multiple instances on Spark executors. In addition, all these applications are created in Java which consumes a lot of memory for initiating each single Java based application needs an extra memory to manage the JVM heap and garbage collections. Initiating multiple instances of these applications is highly inefficient and significantly limits the utility of the solution. This approach is being deprecated for scalability in more recent scalable solutions such as ADAM and VC@Scale. The authors need to discuss such limitations and ways to reduce the memory overhead, such as running multi-threaded versions of these applications, or calling their native functions in Java/Scala code to avoid running multiple instance of these applications on each executor node.

Level of Interest

Please indicate how interesting you found the manuscript: Choose an item.

Quality of Written English

Please indicate the quality of language in the manuscript: Choose an item.

Declaration of Competing Interests

Please complete a declaration of competing interests, considering the following questions:

- Have you in the past five years received reimbursements, fees, funding, or salary from an organisation that may in any way gain or lose financially from the publication of this manuscript, either now or in the future?
- Do you hold any stocks or shares in an organisation that may in any way gain or lose financially from the publication of this manuscript, either now or in the future?
- Do you hold or are you currently applying for any patents relating to the content of the manuscript?
- Have you received reimbursements, fees, funding, or salary from an organization that holds or has applied for patents relating to the content of the manuscript?
- Do you have any other financial competing interests?
- Do you have any non-financial competing interests in relation to this paper?

If you can answer no to all of the above, write 'I declare that I have no competing interests' below. If your reply is yes to any, please give details below.

I declare that I have no competing interests

I agree to the open peer review policy of the journal. I understand that my name will be included on my report to the authors and, if the manuscript is accepted for publication, my named report including any attachments I upload will be posted on the website along with the authors' responses. I agree for my report to be made available under an Open Access Creative Commons CC-BY license (http://creativecommons.org/licenses/by/4.0/). I understand that any comments which I do not wish to be included in my named report can be included as confidential comments to the editors, which will not be published.

Choose an item.

To further support our reviewers, we have joined with Publons, where you can gain additional credit to further highlight your hard work (see: https://publons.com/journal/530/gigascience). On publication of this paper, your review will be automatically added to Publons, you can then choose whether or not to claim your Publons credit. I understand this statement.

Yes Choose an item.
